# Imputation-Based Population Genetics Analysis of *Plasmodium falciparum* Malaria Parasites

**DOI:** 10.1371/journal.pgen.1005131

**Published:** 2015-04-30

**Authors:** Hanif Samad, Francesc Coll, Mark D. Preston, Harold Ocholla, Rick M. Fairhurst, Taane G. Clark

**Affiliations:** 1 Faculty of Infectious and Tropical Diseases, London School of Hygiene and Tropical Medicine, London, United Kingdom; 2 Malawi-Liverpool-Wellcome Trust Clinical Research Programme, Blantyre, Malawi and Liverpool School of Tropical Medicine, Liverpool, United Kingdom; 3 Laboratory of Malaria and Vector Research, National Institute of Allergy and Infectious Diseases, National Institutes of Health, Bethesda, Maryland, United States of America; 4 Faculty of Epidemiology and Population Health, London School of Hygiene and Tropical Medicine, London, United Kingdom; Ospedale San Pietro Fatebenefratelli, ITALY

## Abstract

Whole-genome sequencing technologies are being increasingly applied to *Plasmodium falciparum* clinical isolates to identify genetic determinants of malaria pathogenesis. However, genome-wide discovery methods, such as haplotype scans for signatures of natural selection, are hindered by missing genotypes in sequence data. Poor correlation between single nucleotide polymorphisms (SNPs) in the *P*. *falciparum* genome complicates efforts to apply established missing-genotype imputation methods that leverage off patterns of linkage disequilibrium (LD). The accuracy of state-of-the-art, LD-based imputation methods (IMPUTE, Beagle) was assessed by measuring allelic *r^2^* for 459 *P*. *falciparum* samples from malaria patients in 4 countries: Thailand, Cambodia, Gambia, and Malawi. In restricting our analysis to 86k high-quality SNPs across the populations, we found that the complete-case analysis was restricted to 21k SNPs (24.5%), despite no single SNP having more than 10% missing genotypes. The accuracy of Beagle in filling in missing genotypes was consistently high across all populations (allelic *r^2^*, 0.87-0.96), but the performance of IMPUTE was mixed (allelic *r^2^*, 0.34-0.99) depending on reference haplotypes and population. Positive selection analysis using Beagle-imputed haplotypes identified loci involved in resistance to chloroquine (*crt*) in Thailand, Cambodia, and Gambia, sulfadoxine-pyrimethamine (*dhfr*, *dhps*) in Cambodia, and artemisinin (*kelch13*) in Cambodia. *Tajima’s D*-based analysis identified genes under balancing selection that encode well-characterized vaccine candidates: apical merozoite antigen 1 (*ama1*) and merozoite surface protein 1 (*msp1*). In contrast, the complete-case analysis failed to identify any well-validated drug resistance or candidate vaccine loci, except *kelch13*. In a setting of low LD and modest levels of missing genotypes, using Beagle to impute *P*. *falciparum* genotypes is a viable strategy for conducting accurate large-scale population genetics and association analyses, and supporting global surveillance for drug resistance markers and candidate vaccine antigens.

## Author Summary

Characterizing genetic diversity and function in *Plasmodium falciparum*, including identifying determinants of emerging drug resistance, is crucial to informing public health strategies to contain and eliminate this malaria parasite. The lack of a robust framework to handle missing *P*. *falciparum* genotypes arising from next-generation sequencing efforts, impedes genome-wide methods that depend on complete genotype information, and often leads to analysis that discards entire regions of the genome. This study is the first to evaluate the performance of missing data imputation or “filling in” in the *P*. *falciparum* genome, where the correlation between genetic markers is generally lower than in the human genome. We considered 86k markers in 459 clinical isolates from 4 malaria-endemic populations of Africa and Southeast Asia. Although low genotype missingness per SNP (<10%) results in complete datasets for only 25% of SNPs, imputation is accurate. This finding is corroborated by the ability of imputed haplotype analysis to recover several well-established vaccine candidates and drug resistance loci, including *kelch13*—a recently-validated gene involved in artemisinin resistance. Our work demonstrates that imputation can assist the application of genome-wide methods to identify the determinants of *P*. *falciparum* diversity, including those involved in drug resistance, immune evasion, and host virulence.

## Introduction

Malaria is a major global health burden, with drug resistance in *Plasmodium falciparum* a major impediment to disease containment and eradication. A deeper understanding of the biology of this parasite and the epidemiology of the disease it causes is needed to inform public health responses, including the timely development of new therapeutics and vaccines [1,2]. Interrogating the genetic determinants of *P*. *falciparum* virulence has become a crucial aspect of malaria surveillance and control strategies [2,3]. A large catalogue of high-density single nucleotide polymorphisms (SNPs) in *P*. *falciparum* has been identified through whole-genome sequencing studies, enabling population genetics analysis to characterize global parasite diversity [2,4]. This advance has also facilitated the discovery of putative genetic determinants of drug resistance phenotypes and candidate vaccine antigens [3,5–7]. These and additional SNPs discovered in expanding collections of global *P*. *falciparum* clinical isolates [2,4] may provide new insights into the diversity and pathogenicity of contemporary parasite populations.

The ability to locate naturally-selected regions of this parasite’s genome is critical in uncovering the genetic determinants of drug resistance or candidate vaccines. Several SNPs in *P*. *falciparum* genes are known to confer resistance to antimalarial drugs such as chloroquine (*crt* [8]), sulfadoxine-pyrimethamine (*dhps* [9], *dhfr* [10]), and artemisinin derivatives (*kelch13* [3]). Statistically significant signatures of recent positive selection, such as long-range haplotypes and extended-haplotype homozygosity (EHH), have been used to identify genetic mutations contributing to drug resistance in parasite populations. Similarly, the detection of signatures of balancing selection facilitates the identification of parasite targets of acquired immunity. As immunity to the commonest alleles rises in malaria-endemic areas, rarer alleles confer parasites a selective advantage and correspondingly increase in frequency, thereby displacing previously common alleles. This process maintains a balance of alleles in the population, with neither the common alleles moving to fixation nor the rare alleles moving to extinction. Balancing selection is detected using statistics such as *Tajima’s D* [11] and is evident in vaccine targets, including that encoded by *ama1* [12].

*P*. *falciparum* has a complex life cycle comprising a haploid phase in humans and a diploid phase in *Anopheles* mosquitoes, which involves gamete fertilization and sexual recombination [13]. Genetic variants in the *P*. *falciparum* genome (23 Mb, 14 chromosomes, 81% AT content, ~5500 genes) were initially surveyed using Sanger sequencing on a limited set of laboratory-adapted clones [14,15]. Although recent genomic scans use next-generation sequencing technologies [4,6], high AT content and technological limitations lead to uneven genomic coverage; thus, a proportion of SNP genotypes may be called as missing, which compromises downstream analysis. For example, typical approaches exclude SNPs and parasite isolates with high rates (>10%) of missingness from subsequent analysis [4]. Harnessing the full potential of fine-scale population genetics methods, such as haplotype scans for signatures of natural selection, requires complete genotype data on a dense set of SNPs in a large number of samples. The standard approach of restricting analysis only to SNPs without missing data may be problematic. Low levels of individual SNP missingness, for example, can result in small numbers of SNPs with complete genotypes across all samples, leading to loss of statistical power and introduction of potential bias.

Imputation methods that reconstruct haplotypes using linkage disequilibrium (LD) patterns have become routine ways of handling missing genotypes in human genome-wide studies [16–19]. However, these methods may be compromised in low LD settings, where high rates of recombination in *P*. *falciparum* lead to decay in inter-SNP correlation within 500 bp [2,16]. Validating the imputation of missing genotypes in whole-genome sequences could enable population genetics methods to scale more appropriately with increasing sequences and SNPs, without having to resort to customized missing data strategies. Here we present a novel evaluation of LD-based imputation applied to a large number of global *P*. *falciparum* samples and demonstrate its utility in population genetics analysis. Two state-of-the-art methods, IMPUTE [17] and Beagle [18], were assessed by masking known genotypes and measuring the accuracy of imputed genotypes using an allelic *r* ^*2*^ statistic [18]. Briefly, IMPUTE is based on the LD framework of Li and Stephens [19], with the haplotype to be imputed modelled as a sampled path through all possible underlying states defined by a hidden Markov model (HMM) constructed from a set of complete haplotypes. Externally inferred recombination rates determine the transition probabilities from one haplotype position to the next, while a mutation rate parameter controls the emission probabilities determining fidelity from hidden to observed states. Beagle constructs an HMM by clustering observed haplotypes at each marker position such that hidden states correspond to groups of haplotypes [18], with each group representing haplotypes with similar transition probabilities for nearby downstream alleles. Recombination is implicit within the modelled transition probabilities and no mutation parameter is incorporated.

We took advantage of the structure of IMPUTE software to test LD-based recombination rates derived from 2 high-quality parasite genetic crosses (7G8-Brazil × GB4-Ghana [20]; DD2-Indochina × HB3-Honduras [21]) and a coalescent-based model of LD in LDhat software [22]. This was complemented by evaluating the empirical model of LD in Beagle software based on its haplotype clustering algorithm [18].

By completing multiple SNP datasets using these methods, we performed genome-wide scans for signatures of natural selection using EHH-type [23] and *Tajima’s D* [11] metrics, and a complementary analysis of parasite population structure. *P*. *falciparum* populations are geographically dispersed and undergo regional adaptation due to selection pressures from antimalarial drugs, and human and mosquito immune responses. The effect of imputation on patterns of population structure was assessed using principal components analysis (PCA) and pairwise population *Fst* [24]. All results were compared to a “complete-case analysis,” where SNPs with missing genotypes were excluded. We demonstrate that currently available LD-based imputation methods used previously in human populations can be extended to sequence data from *P*. *falciparum* populations, with imputed genotypes reliably capturing known and potentially novel loci encoding drug resistance phenotypes and candidate vaccine antigens.

## Results

### *P*. *falciparum* SNP datasets

Sequencing reads from 2 Southeast Asian (Thailand, n = 91; Cambodia, n = 253) and 2 African (Gambia, n = 55; Malawi, n = 60) populations of *P*. *falciparum* clinical isolates [2] were aligned to the *P*. *falciparum* 3D7 reference genome (version 3) [25]. A total of 85,967 biallelic SNPs (“86k set”) across these 459 isolates were identified, representing a marker density of 1 SNP every 270 bp. The 86k set represents 4,799 genes (SNPs per gene: median, 4; 90^th^ percentile, 17) and was dominated by markers that are monomorphic in at least 1 population (Thailand, 50,087 (58%); Cambodia, 34,049 (40%); Gambia, 53,539 (62%); Malawi, 50,287 (59%)). The Southeast Asian populations share 26,551 (31%) SNPs while the African populations share 24,089 (28%) SNPs. Only 7,855 (9.1%) SNPs are shared between all populations.

**Fig 1:**
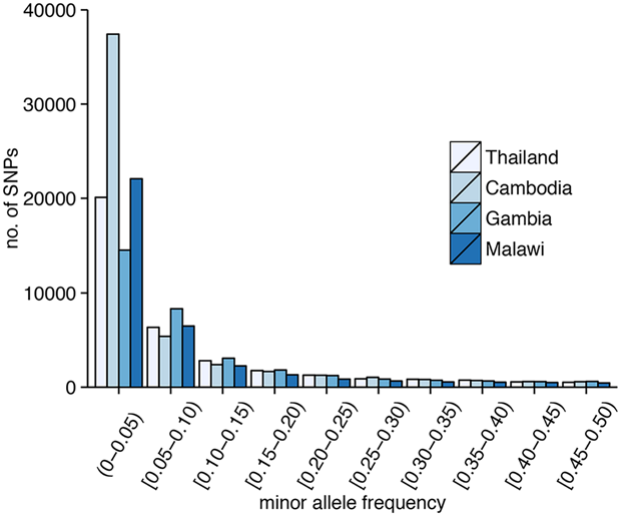
Distribution of SNPs according to minor allele frequency (MAF). In all parasite populations, there is an overabundance of low-frequency SNPs (MAF<5%).

**Fig 2:**
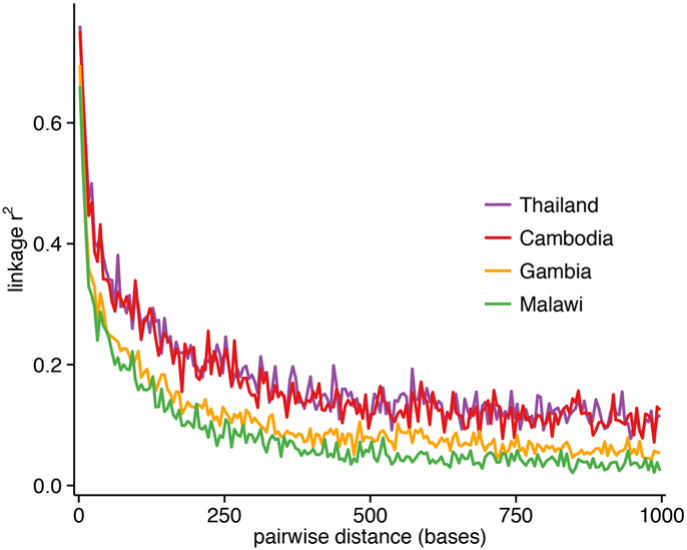
Linkage disequilibrium (LD) decay as a function of genomic distance in each population, using *r* ^*2*^ between pairs of markers with MAF≥5% and within 1000 bp of each other. The plotted *r* ^*2*^ is the mean calculated for pairwise distances binned in 5-bp increments. LD decays to *r* ^*2*^ = 0.2 within approximately 187 bp for Cambodia, 162 bp for Thailand, 102 bp for Gambia, and 67 bp for Malawi.

Excluding SNPs that are monomorphic in each population, low-frequency SNPs predominate in the 86k set (minor allele frequency (MAF)<5%: Thailand, 62%; Cambodia, 72%; Gambia, 45%; Malawi, 59%) (Fig 1), and the overall MAF is low (median 0.8%; 90^th^ percentile 11%). Using common SNPs (MAF≥5%), there was evidence that LD decayed rapidly within a few hundred base pairs in the African populations, and reached a baseline within 500 bp. Compared to the Southeast Asian populations, LD decayed more rapidly in the African populations (Fig 2), consistent with their higher recombination rates (S1 Table). The estimated recombination rates are especially high in Malawi, resulting from high levels of outcrossing associated with relatively high transmission intensity and multiplicity of infection [6].

Isolates and SNPs had less than 10% of their genotypes missing, reflecting the current practice of analysing only high-quality samples (median, 0.3%; 90^th^ percentile, 3.1%; maximum, 8.9%) and markers (median, 0.4%; 90^th^ percentile, 2.4%; maximum, 8.3%). The number of SNPs with complete genotypes varied by population (Thailand, 56,797 (66%); Cambodia, 47,482 (55%); Gambia, 53,469 (62%); Malawi, 54,419 (63%); Table 1). Restricting analysis to those SNPs with complete genotypes across the 459 samples led to severe data depletion, reducing the number of SNPs from 85,967 to 21,077 (24.5%). A strong positive correlation between SNP-wise rate of missingness and MAF (S1 Fig) would have excluded most common SNPs from a complete-case analysis of these 21,077 SNPs (MAF: median, 0.4%; 90^th^ percentile, 1.5%). This correlation could be an artefact of genotype calling from sequencing reads. In particular, because the genotypes were called using ratios of coverage, genotypes from SNPs with a higher minor allele frequency are less likely to pass the calling threshold due to more balanced coverage ratios. If the threshold was lowered, more of those genotypes are likely to be called erroneously.

**Table 1:** The accuracy and minor allele concordance rate of imputed genotypes.

Population	n	% of 86k SNPs that are polymorphic	% SNPs with any missing data	Imputation method (recomb. rate source)	Refer. haplo.	Mean *r* ^*2*^ by SNP	Mean minor allele concord. (%)
**Thailand(THL)**	91	42%	34%	IMPUTE2	90 THL	0.51	31.8
				(GB4x7G8)	471 ALL	0.70	66.5
				IMPUTE2	90 THL	0.53	38.2
				(HB3xDD2)	471 ALL	0.70	67.2
				IMPUTE2	90 THL	0.56	46.1
				(LDhat)	471 ALL	0.71	70.7
				BEAGLE	90 THL	0.92	91.4
				(map free)	471 ALL	0.27	29.0
**Cambodia (CBO)**	253	60%	45%	IMPUTE2	252 CBO	0.77	68.8
				(GB4x7G8)	471 ALL	0.87	82.5
				IMPUTE2	252 CBO	0.78	73.7
				(HB3xDD2)	471 ALL	0.88	83.2
				IMPUTE2	252 CBO	0.84	79.1
				(LDhat)	471 ALL	0.90	86.1
				BEAGLE	252 CBO	0.96	92.1
				(map free)	471 ALL	0.65	63.3
**Gambia (GMB)**	55	38%	38%	IMPUTE2	54 GMB	0.99	95.4
				(GB4x7G8)	471 ALL	0.99	95.2
				IMPUTE2	54 GMB	0.99	95.4
				(HB3xDD2)	471 ALL	0.99	95.4
				IMPUTE2	54 GMB	0.99	95.4
				(LDhat)	471 ALL	0.99	95.4
				BEAGLE	54 GMB	0.90	87.1
				(map free)	471 ALL	0.21	23.0
**Malawi (MLW)**	60	41%	37%	IMPUTE2	59 MLW	0.34	9.5
				(GB4x7G8)	471 ALL	0.25	20.2
				IMPUTE2	59 MLW	0.30	9.2
				(HB3xDD2)	471 ALL	0.25	20.8
				IMPUTE2	59 MLW	0.69	49.0
				(LDhat)	471 ALL	0.26	12.3
				BEAGLE	59 MLW	0.87	85.0
				(map free)	471 ALL	0.06	6.7

Accuracy is consistently high with Beagle when only population-specific haplotypes are used as references; improves with IMPUTE when a cosmopolitan reference panel is used; and is highest with IMPUTE when LDhat-inferred recombination rates are used. ALL refers to a cosmopolitan panel including populations from the listed countries (n = 459) and Vietnam (n = 12).

### Imputation accuracy

SNPs in chromosome 13 were chosen to compare imputation strategies, since this is the second-to-longest chromosome containing correspondingly large numbers of SNPs, and it contains the *kelch13* locus recently associated with artemisinin resistance in Southeast Asia [3]. Using a leave-one-out cross-validation approach, marker genotypes were masked and the accuracy of imputed calls to the true allele was measured using an allelic correlation metric *r* ^*2*^ (Table 1). The imputation accuracy of Beagle was consistently high (mean *r* ^*2*^, 0.87–0.96), reaching levels seen in a human genome-wide setting [19]. However, there was substantial variation in IMPUTE results across populations, with near-perfect mean *r* ^*2*^ values in Gambia (0.99) and high values in Cambodia (0.77–0.84), contrasting with lower values in Thailand (0.51–0.56) and Malawi (0.30–0.69). Using a cosmopolitan panel of reference haplotypes from all 4 populations (including 12 Vietnamese samples with complete genotypes), the accuracy of IMPUTE was improved in Thailand (mean *r* ^*2*^, 0.70–0.71) and Cambodia (0.82–0.90) but degraded in Malawi (0.25–0.26). Using the same cosmopolitan panel, the accuracy of Beagle was substantially degraded in all 4 populations including Thailand (mean *r* ^*2*^, 0.27). For IMPUTE, recombination rates inferred from LDhat were marginally more accurate than those inferred from analysing 2 genetic crosses. In Malawi, accuracy was substantially improved when using a reference panel specific to this population (mean *r* ^*2*^, 0.30–0.69). Using a lower mutation rate parameter (*θ* = 0.001) or effective population size (*N*_*e*_ = 10,000) led to only marginal improvements in accuracy in the Thai (mean *r* ^*2*^, 0.62–0.64), but not Gambian (mean *r* ^*2*^, 0.99) population (S2 Table). Rates of minor allele concordance, defined as the agreement between absolute genotype calls (made on a threshold of ≥0.9 on posterior probabilities) and true minor alleles, tracked the measured *r* ^*2*^ closely (Pearson’s correlation = 0.75).

**Fig 3:**
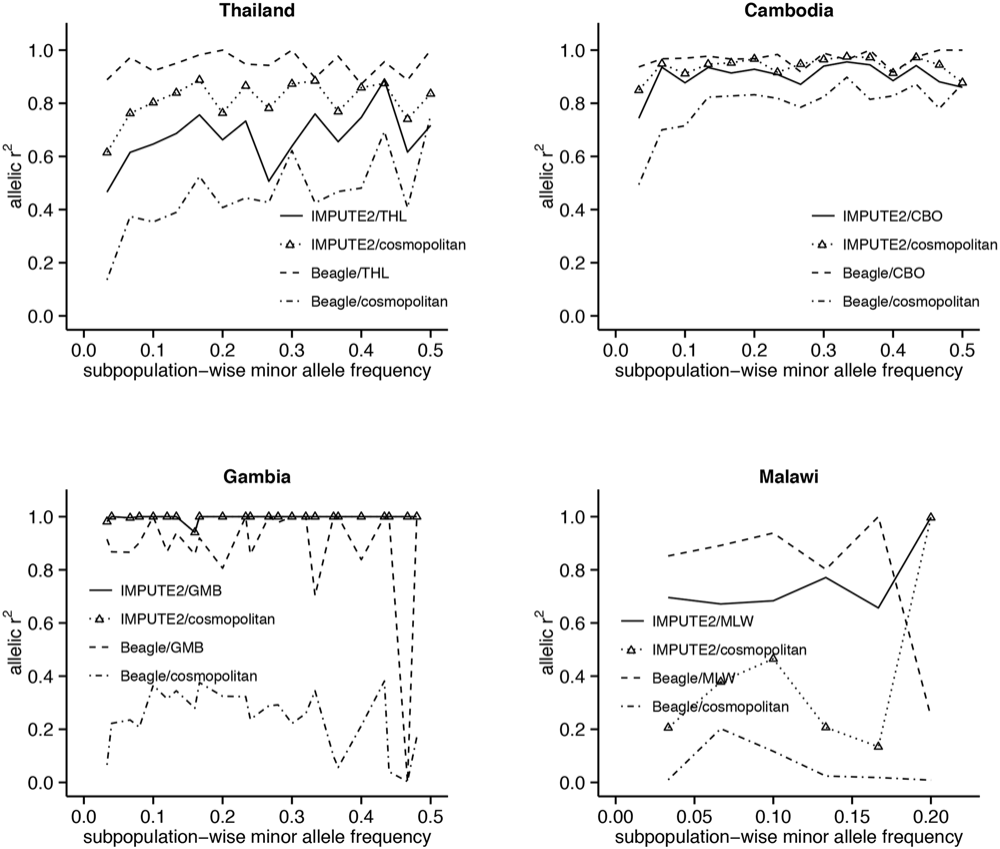
Relationship between imputation accuracy and minor allele frequency (MAF), tested on chromosome 13 SNPs. MAF-wise means are used to infer the trend in *r* ^*2*^. Only designs using LDhat-inferred recombination rates are considered for IMPUTE. Use of a cosmopolitan reference panel improves accuracy across both common and low-frequency SNPs in IMPUTE; however, accuracy decreases substantially in Beagle. SNPs in Malawi have a maximum MAF = 0.23.

The accuracy of LD-based imputation increases with MAF in the Southeast Asian populations, with most improvement occurring in the low-frequency range (Fig 3). This increasing trend is also observed in studies imputing human genotypes [16]. In Gambia, the accuracy of Beagle is generally high except for one SNP at MAF 47% with *r* ^*2*^~0. In contrast, the near-perfect accuracy of IMPUTE in Gambia is maintained across the allele-frequency spectrum. In Malawi, because of the limited range (3–23%) of MAF values and paucity of SNPs with higher frequencies, a trend for *r* ^*2*^ could not be clearly discerned in any of the imputation designs. Across all designs there was no systematic change in MAF post-imputation (S2 Fig).

Cross-validation experiments indicated that the optimal imputation accuracy of Beagle occurs when a population-specific reference panel is used, and that for IMPUTE occurs when LDhat-derived recombination rates and a cosmopolitan reference panel are used. For each imputation design, a set of 10 SNP datasets was completed by filling in missing data using sampling of posterior genotype probabilities (Materials and Methods). To assess the effect of imputation strategies, we focused on selection metrics that require complete SNP datasets, namely those that detect long-range haplotypes and extended-haplotype homozygosity, both within (|*iHS*|) and between (*Rsb*, a version of *XP-EHH* [26]) populations. These metrics were computed on each dataset, and results pooled using a multiple imputation approach to provide overall estimates. Pearson’s *r* correlation of the selection metrics between Beagle- and IMPUTE-derived haplotypes was very high across the 4 populations, for both |*iHS*| (range, 0.93–0.98) and *Rsb* (range, 0.98–0.99, using Malawi as the reference population). IMPUTE-derived haplotypes systematically resulted in higher *Rsb* values compared to Beagle-derived haplotypes (S3 and S4 Figs). The correlation between selection metrics from Beagle-imputed versus complete-case genotypes was much lower (e.g., |*iHS*| *r*<0.60; S5 and S6 Figs). Given the arguably superior performance of Beagle and its ease of use, we proceeded by comparing the top selection hits (overall 1% of threshold across populations, |*iHS*|>2.45, *Rsb*>4) between Beagle-imputed and complete-case haplotype analyses.

### Recent positive selection

**Fig 4:**
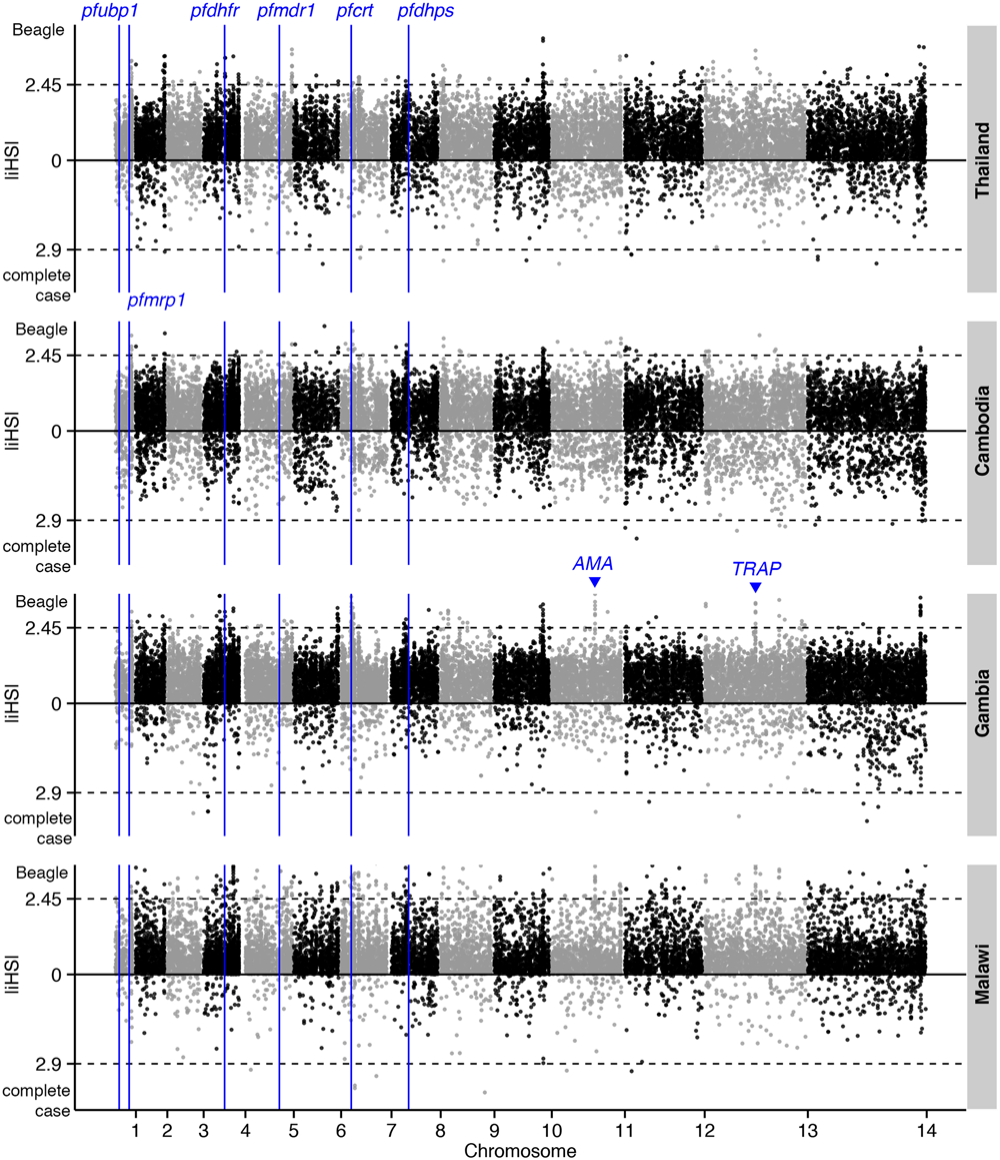
The top |*iHS*| hits in 4 global *P*. *falciparum* populations. Dashed lines indicate the top 1% of |*iHS*| values (median threshold across 4 populations) for Beagle-imputed (above zero) and complete-case (below zero) data. The positions of genes known to confer drug resistance or encode vaccine candidates are annotated.

**Fig 5:**
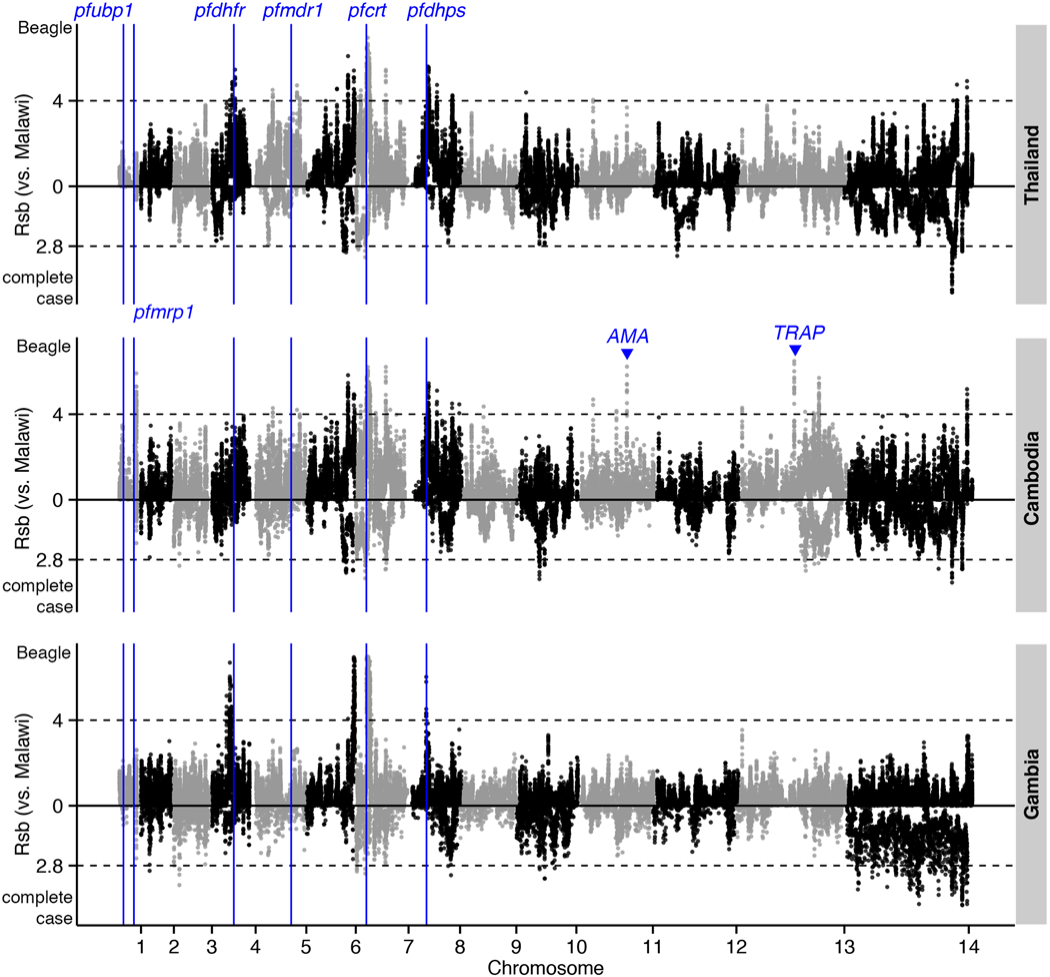
The top *Rsb* hits for Thailand, Cambodia, and Gambia, using Malawi as the reference population. Dashed lines indicate the top 1% of *Rsb* values (median threshold across 3 populations) for Beagle-imputed (above zero) and complete-case (below zero) data.

Evidence for selective sweeps due to drug pressure or other mechanisms was investigated using the |*iHS*| and *Rsb* metrics applied to Beagle-imputed haplotypes. Intra-population analysis revealed signals downstream of *crt*, including *cg1* and *cg2*, in the African populations, but not in the Southeast Asian populations in which mutant alleles are already fixed (Fig 4 and S3 Table). Other strong |*iHS*| hits included genes encoding vaccine candidates (e.g., *ama1* and *trap*) and other membrane and surface proteins (e.g., *clag2* and *msp4*). In contrast, analysis of complete-case haplotypes only identified *ama1* (S4 Table). Inter-population analysis using the *Rsb* metric has the potential to detect positively-selected alleles that have already achieved fixation. With Malawi as the reference population, *crt* and neighbouring *cg1* and *cg2* were strongly identified across the other 3 populations (Fig 5 and S5 Table). Whilst sulfadoxine-pyrimethamine resistance genes were not directly identified, there were strong signals in regions proximal to *dhfr* (e.g., *PF3D7_0417400*, S7 Fig) and *dhps* (e.g., *PF3D7_0809800*, S8 Fig) in Southeast Asian populations. This phenomenon of signal ‘shifting’ is due to the beneficial allele sweeping up in parallel in the Malawian reference population, giving rise to long-range haplotypes that attenuate the inter-population differences in the EHH at regions close to the SNP conferring drug resistance. *Rsb* analysis also confirmed positive selection in *ama1* and *trap* in Southeast Asian populations.

**Fig 6:**
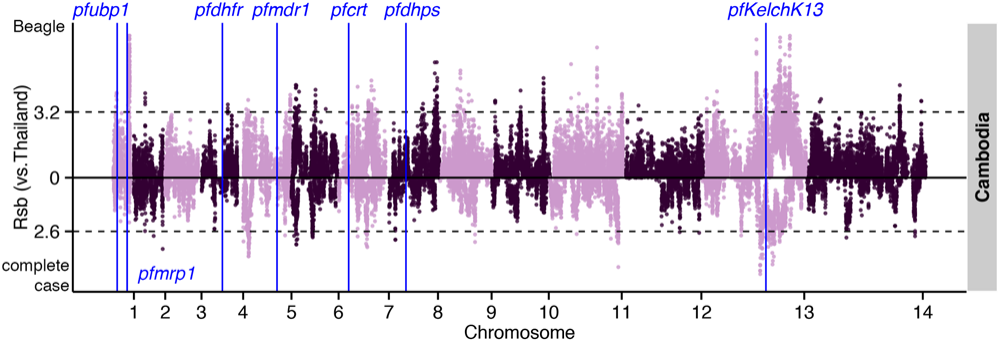
The top *Rsb* hits for Cambodia using Thailand as the reference population. Dashed lines indicate the top 1% of *Rsb* values for imputed (above zero) and complete-case (below zero) data.

Analysis of complete-case haplotypes failed to detect *crt*, *dhfr*, and *dhps* effects (S6 Table). *Rsb* analysis of these haplotypes did, however, confirm positive selection in *ama1* and *trap* in Southeast Asian compared to Malawian populations. Importantly, this analysis also identified positive selection in *kelch13* [3] in Cambodian compared to Thai populations (S7 Table), as well as in 2 other loci: a ~600kb region downstream of *kelch13* (Fig 6); and *PF3D7_0104100*, in a region upstream of *pfubp1* [5], detected previously in a selecton scan of Kenyan parasites [5]. In this instance, analysis of complete-case haplotypes also managed to identify a signal in *kelch13* (S7 Table), indicating that the sweep is young and the signal has not yet been lost through recombination. The complete-case haplotype analysis also detected *pfs45/48* and *pfs47* that mediate evasion of the mosquito immune system, and are a signature of highly-structured populations resulting from inter-continental differences in the prevalence of *Anopheles* vector species [27].

### Balancing selection

Using Beagle-imputed haplotypes, *Tajima’s D* was calculated by population for each of the 3,517 (of 4,799) genes with 3 or more SNPs. The vast majority of *Tajima’s D* values were negative (Thailand, 85%; Cambodia, 89%; Gambia, 86%; Malawi, 93%), indicating a demographic history of recent population expansion [6,28,29] or purifying selection. The malaria parasite life cycle itself can also lead to a skewed frequency spectrum toward low frequency alleles and negative *Tajima’s D* [29]. Amongst the genes with high *Tajima’s D* values (>1) based on at least 10 SNPs (Table 2), several encode well-described targets of acquired immunity such as *ama1* (all populations, 1.16–1.79) and *msp1* (Southeast Asia, 2.23–2.29). Other genes previously found to have high *Tajima’s D* values [7] were also identified, including *eba175* (all populations, 1.02–1.55), *dblmsp* (non-Malawi, 1.22–2.11), and *clag2* (non-Malawi, 1.35–1.71). Both *ama1* and *msp1* have been previously identified to be under positive or balancing selection in certain parasite populations [5,29]. Using the set of unimputed SNPs, nearly all of the 3,517 *Tajima’s D* values were negative (Thailand, 99.5%; Cambodia, 100%; Gambia, 99%; Malawi, 100%), reflecting the exclusion of common SNPs and inability to calculate pairwise nucleotide differences between haplotypes in a population with missing genotypes. The exclusion of common SNPs may also lead to an overestimate of population expansion.

**Table 2:** Genes (n = 62) with ≥10 SNPs having *Tajima’s D* (TD) values >1 in populations from Thailand (total 793 genes), Cambodia (1208 genes), Gambia (723 genes), and Malawi (812 genes).

Chr	Gene ID	Gene name	Thailand (TD)	Cambodia (TD)	Gambia (TD)	Malawi (TD)	Median no. of SNPs
1	PF3D7_0103600		-	-	1.42	-	23
1	PF3D7_0104100		1.07	-	-	-	16
1	PF3D7_0113600	*SURFIN1*.*2*	-	1.38	-	-	96
1	PF3D7_0113800		1.82	2.07	1.46	1.03	134
2	PF3D7_0202100	*LSAP2*	1.39	-	-	-	10
2	PF3D7_0207000	*MSP4*	-	1.12	-	-	11
2	PF3D7_0211700	*TKL1*	1.28	-	-	-	21
2	PF3D7_0215300	*ACS8*	-	-	1.19	-	18
2	PF3D7_0220000	*LSA3*	-	-	1.12	-	16
2	PF3D7_0220800	*CLAG2*	1.71	1.16	1.35	-	54
2	PF3D7_0220900		-	2.81	1.14	-	10
2	PF3D7_0221000		-	1.02	1.44	1.76	12
3	PF3D7_0318200		-	1.01	-	-	21
3	PF3D7_0319200		-	2.12	-	-	10
4	PF3D7_0412300		1.77	1.77	1.20	1.46	12
4	PF3D7_0418000		1.23	-	1.43	-	32
4	PF3D7_0420200		-	-	2.72	1.84	14
4	PF3D7_0424900		1.88	-	-	-	11
4	PF3D7_0425000		1.61	1.06	-	-	27
5	PF3D7_0503200		-	-	1.11	-	11
5	PF3D7_0511300		-	-	1.33	-	26
5	PF3D7_0511400		-	-	1.11	-	10
5	PF3D7_0516300		-	2.23	2.19	1.39	14
5	PF3D7_0519300		-	-	1.10	-	12
5	PF3D7_0525100	*ACS10*	-	-	1.20	-	11
5	PF3D7_0532300		1.17	1.19	1.09	-	14
7	PF3D7_0704600		1.46	-	-	-	33
7	PF3D7_0707200		1.32	-	-	-	12
7	PF3D7_0708400	*HSP90*	-	-	-	1.06	15
7	PF3D7_0709300	*CG2*	-	-	-	1.39	55
7	PF3D7_0710000		-	-	1.28	-	60
7	PF3D7_0711200		1.74	-	-	-	13
7	PF3D7_0711500		1.06	1.31	1.26	-	24
7	PF3D7_0713900		1.01	-	-	-	57
7	PF3D7_0720400		-	-	1.60	1.63	10
7	PF3D7_0729700		-	-	3.17	2.71	10
7	PF3D7_0731500	*EBA175*	1.34	1.26	1.55	1.02	25
7	PF3D7_0731800	*GEXP08*	-	1.53	-	-	14
8	PF3D7_0808200		1.68	2.99	-	-	10
8	PF3D7_0809700	*RUVB1*	-	-	1.52	1.13	12
8	PF3D7_0812900		1.70	-	-	-	13
8	PF3D7_0822700		1.01	-	-	-	11
8	PF3D7_0824400	*NT2*	-	-	2.38	-	10
9	PF3D7_0905600		-	-	2.14	1.36	15
9	PF3D7_0926500		1.07	-	-	-	16
9	PF3D7_0930300	*MSP1*	2.23	2.29	-	-	28
9	PF3D7_0931300		2.35	-	-	-	10
10	PF3D7_1002200	*PArt*	-	-	1.55	1.25	13
10	PF3D7_1034900			1.51	-	-	13
10	PF3D7_1035000			1.60	-	-	35
10	PF3D7_1035700	*DBLMSP*	1.82	2.11	1.20	-	22
11	PF3D7_1110200	*PRPF6*	-	-	2.18	1.92	11
11	PF3D7_1126100	*ATG7*	1.49	-	1.43	-	15
11	PF3D7_1133400	*AMA1*	1.36	1.79	1.16	-	30
11	PF3D7_1145500		1.21	-	-	-	10
12	PF3D7_1208200	*CRMP3*	2.02	1.27	-	-	19
13	PF3D7_1302900		-	1.24	2.15	1.86	11
13	PF3D7_1320700		-	1.06	-	-	15
13	PF3D7_1335100	*MSP7*	-	1.33	-	-	13
14	PF3D7_1429800		-	-	1.16	-	18
14	PF3D7_1475800		-	1.31	-	-	27
14	PF3D7_1475900		1.41	2.25	-	-	34

Median TD values obtained from 10 imputed datasets are shown.

### Population structure

Principal components analysis using both unimputed or imputed data revealed similar well-defined population structure, namely clustering firstly at a continental level (S9 Fig), and secondarily at regional and population levels. The extent to which SNPs drive these inter-population differences was quantified using the *Fst* differentiation metric. As expected, greater mean *Fst* values were attained between (range: unimputed, 0.260–0.273; imputed, 0.259–0.267) than within (range: unimputed, 0.035–0.057; imputed, 0.040–0.058) continents (S8 Table). As the *Fst* implementation was SNP- rather than haplotype-based, there was little appreciable difference between unimputed and imputed results (P>0.05), further supporting the robustness of population structure analysis of datasets with low frequencies of missing genotypes.

## Discussion

The ability to monitor for signatures of selection in *P*. *falciparum* is being revolutionised by whole-genome sequencing of large numbers of global clinical isolates. One unintended consequence of this effort, however, is that sequencing greater numbers of parasite genomes will increase the number of missing genotypes, presenting new challenges to haplotype-based population genetics analysis that requires complete SNP datasets. Imputation approaches have been successfully applied to human genomes, but high rates of recombination and rapid LD decay challenge their application to *P*. *falciparum* genomes. To help meet these challenges, our study shows that readily available software (Beagle and IMPUTE) produce accuracy levels comparable to those in human studies, and identify known *P*. *falciparum* loci involved in drug resistance and immune evasion. Application of Beagle with a population-specific panel of reference haplotypes provided consistently high levels of imputation accuracy across 4 parasite populations, suggesting that its estimation of local haplotype clusters reliably reproduces LD structure in each population. In contrast, accuracy with IMPUTE varied between populations using a similar population-specific referencing strategy.

Recombination maps were derived from genetic crosses of parental strains from Honduras, Indochina, Brazil, and Ghana. These are unlikely to represent haplotype configurations that accurately characterise LD structure in other populations, and therefore led to lower accuracy of samples from geographically-distant countries (e.g., Thailand) in our study. Inferring recombination maps from population haplotypes via LDhat marginally improved the performance of IMPUTE. There were differences in recombination rates between inferred and experimentally derived maps (S10 Fig). The near-perfect accuracy in Gambian samples observed for IMPUTE was replicated consistently across different recombination maps, due in part to lower levels of missing genotypes, but this was the exception. Adopting an ancestrally-diverse reference or cosmopolitan panel within IMPUTE, however, significantly increased accuracy across the allele-frequency spectrum. Using a cosmopolitan panel unconstrains the IMPUTE algorithm to choose the most similar set of haplotypes, including those where the parasites of origin may be genealogically distant [30]. However, use of such a panel worsened Beagle’s performance in all populations, due in part to its less flexible haplotype clustering and LD modelling on all haplotypes supplied [18].

While useful to test the validity of LD models via recombination maps derived from different sources, the design of the IMPUTE software is such that LD is modelled independently of missing genotypes using a complete reference panel [17], suggesting that the sporadic missingness of sequencing data may be problematic to the construction of such a model. In addition, the necessity of many free parameters required as inputs made it a less robust imputation strategy for *P*. *falciparum*. Newer methods of LD inference such as LDhelmet [31] may improve the underlying model of recombination; however, IMPUTE is intrinsically not very amenable to imputing sporadically missing genotypes. In contrast, Beagle infers LD by jointly modelling the missing and observed genotypes. Rather than making hard genotype calls, genotype probabilities inferred from the Beagle and MACH [32] methods could improve the handling of uncertainty at the SNP discovery phase and potentially enable more SNPs to be imputed and used in downstream analyses.

The top gene hits using EHH-based metrics computed from imputed haplotypes suggest that imputation is successful in reproducing signatures of positive selection, even when large numbers of haplotypes with initially extensive missingness are used. As in previous haplotype scans for signatures of positive selection in *P*. *falciparum* [5,33–36], we confirmed the *crt* signal using the inter-population metric *Rsb*. Signals from the intra-population metric |*iHS*| (with MAF≥5%) were likely to miss evidence of near or full selective sweeps, as in Southeast Asia where beneficial *crt* alleles and their associated long-range haplotypes have reached fixation. More-recent selective sweeps were observed in regions close to the sulfadoxine-pyrimethamine (SP) resistance genes *dhfr* and *dhps* in the Thai, Cambodian, and Gambian populations. Unlike chloroquine resistance that spread out of Southeast Asia, SP resistance arose independently across continents. The *Rsb* metric highlighted the known independent sweep in *dhps* in Malawi, leading to signal ‘shifting’ in other populations. This metric also identified the artemisinin resistance gene *kelch13* within a ~600kb region in Cambodian parasites, with Thai parasites as the reference population. This study is the first to detect *kelch13* using a phenotype-independent, whole-genome scan for signatures of positive selection. Since *kelch13* mutations have evolved only recently, this finding suggests that global surveillance of whole-genome *P*. *falciparum* sequence data may successfully detect entirely new forms of resistance to future drugs and vaccines.

Analysis of complete-case haplotypes also detected the *kelch13* signature of selection, likely because this gene had relatively high coverage (1.5-fold greater than the genome average). However, these haplotypes could not validate known drug resistance loci and, overall, their correlation with Beagle-imputed results was low. The absence of known loci was primarily due to exclusion of disproportionately more-common SNPs with missing genotypes. EHH-based methods depend on accurately measuring LD decay around a core SNP. There was a trade-off between inflation of EHH due to lower allele frequencies, and deflation of EHH due to a 75% reduced marker density (i.e., the closest markers are less likely to be in LD). A shift in allelic-frequency spectra towards rare alleles inflates negative *Tajima’s D* values, indicating directional selection. By applying a *Tajima’s D* approach to the imputed data, genes under balancing selection as putative antigenic determinants were identified. For example, *ama1*, *msp1*, and *msp7* encoding well-characterized parasite antigens were detected in multiple populations, although *msp3*.*8* and *trap* previously detected in Malawi [6] and Gambia [37], respectively, did not exceed the stringent *Tajima’s D* threshold of 1. Complete-case genotypes failed to produce robust hits, as ≥99% of *Tajima’s D* values were negative across all populations. The skew towards low-frequency SNPs in the complete-case dataset biases the nucleotide diversity and therefore the *Tajima’s D* statistic. Similarly, the effective sample size is smaller than the analysable sample size after imputation, and may bias variation estimation and *Tajima’s D*. Some loci thought to be under balancing selection were found to be under recent positive selection, highlighting that some loci may be under dual selective pressures depending on where and when parasite samples are collected. Although such loci have signatures similar to incomplete sweeps of positive selection [38], evidence for dual selection pressures on *ama1* and *trap* corroborated similar findings in other populations [5,18].

In summary, we have outlined a viable strategy for imputing missing genotypes in large numbers of global *P*. *falciparum* isolates, taking into account differences in LD and population structure. Specifically, we recommend using Beagle as the preferred algorithm for imputing whole-genome sequences, and population-specific haplotypes as references. This approach may be useful for imputing genome data from other *Plasmodium* spp., with resultant datasets being more complete, better able to identify evidence of natural selection by antimalarial drugs and host immunity, and more likely to lead to new insights and applications for disease control.

## Materials and Methods

### *P*. *falciparum* SNPs and samples

Sequencing reads from 3 Southeast Asian (Cambodia, n = 253; Thailand, n = 91; Vietnam, n = 12) and 2 African populations (Gambia, n = 55; Malawi, n = 60) of *P*. *falciparum* clinical isolates [2] were aligned to the *P*. *falciparum* 3D7 genome (version 3) using *smalt* (described in [2]). For all samples the average coverage across the whole genome was at least 35-fold. Variants were called using *samtools* [39] and *vcftools* [40] with default settings. Genotypes at SNP positions were called using ratios of coverage, with heterozygous calls converted to the majority genotype on a 70:30 or greater coverage ratio [4]. SNPs that reflected possible multiplicity of infection were removed as the imputation methods under evaluation cannot tolerate haploid and mixed genotypes simultaneously. Low-coverage *var* and subtelomeric regions (S9 Table) were discarded. The 85,967 biallelic and non-singleton SNPs with less than 10% missing genotypes were retained, which is approximately the inflection point beyond which SNPs experience a rapid increase in missingness (S11 Fig). Allowing more missing data by relaxing the threshold would lead to a decrease in imputation accuracy [17].

### Cross-validation experiments for imputation accuracy

Performance of the imputation methods was evaluated with a leave-one-out cross-validation approach using markers on chromosome 13, where 215 (~2%) SNPs were masked in each haplotype and imputed using the others as references. This procedure produces an accuracy measure based on the squared Pearson correlation (allelic *r* ^*2*^) between the true and imputed allele probability for each marker [18]. To prevent sampling bias from choosing only markers with completely known genotypes, each population panel was split into several subpopulations of 25–30 isolates each, and independent rounds of cross-validation were performed on a distinct set of markers in each subpopulation. This procedure introduced representation of markers at varying levels of missingness, and increased the total number of validated markers. A concordance rate between true and best-guess genotypes on a calling threshold ≥0.9 was also calculated. Only the concordance of minor alleles is considered to mitigate the possibility of near-monomorphic SNPs inflating overall concordance rates.

### IMPUTE and Beagle

Imputation software established primarily for human genotype analysis were adapted to enable their application to parasite sequence data. Beagle v3.3 [18] does not require recombination rates to inform its LD model and processes sporadically missing genotypes, but only imputes diploid genotypes. Therefore, we duplicated all positions to make sequences homozygous diploid before each imputation round, then heterozygous imputed genotype probabilities were allocated equally between the 2 alternative alleles when computing allelic dosage. For IMPUTE v2.0, controls allowing haploid imputation (-*chrX*, -*use_prephased_g*, -*known_haps_g)* were activated. Previously published recombination maps from 2 high-quality genetic crosses between laboratory clones GB4x7G8 [6,21,38,41] and HB3xDD2 [6,22,38,41] were used to supply 2 sets of experimentally informed rates. These two maps were considered separately, and not combined. An alternative set of population recombination rates (2*N*_*e*_ *r*) was inferred using the coalescent-based LDhat program, *interval* [42]. Pre-supplied likelihood lookup files with a population mutation rate (*θ*) value of 0.01 were used. The effective population size (*N*_*e*_ = 30,000) was derived by re-scaling the compound map distance for Gambian isolates inferred from *interval*, to match the empirical map distance estimated from the GB4x7G8 cross. The scaling factor was treated as a pseudo-*N*_*e*_ used to adjust compound rates uniformly on the other populations. To test the robustness of assuming the above parameter values for *θ* and *N*_*e*_, we computed accuracy using different values for one African (Gambia) and one Southeast Asian (Thailand) population (S2 Table).

### Population genetics metrics

Multiple haplotype sets were sampled from the marginal posterior genotype distribution under the best-performing imputation designs. This procedure allowed the computation of population genetics metrics under a multiple imputation-like schema, accounting for uncertainty in the true identity of genotypes. Ten SNP datasets were completed for each imputation strategy through sampling from posterior genotype probabilities, and relevant population genetics metrics at each SNP or gene were computed and averaged across the sets. Two metrics were used to infer recent positive selection, the integrated haplotype score (*iHS)* and *Rsb*. *iHS* is the standardized log ratio of the integrated extended-haplotype homozygosity (EHH) between the ancestral and derived allele at a core SNP [43], capturing evidence of unusually long haplotypes surrounding a particular allele within the population. The ancestral and derived alleles were defined to be the major and minor allele at Malawian SNPs, as this population is ancestrally ancient [6,16] and the rapid LD decay made it a natural reference point for detecting extended haplotype homozygosity in other populations. Absolute values of *iHS* were used to capture long haplotypes centred around either type of allele, using a minimum MAF filter of 5%. *Rsb* is the standardized log ratio of the integrated site-specific EHH between populations at a core SNP, where site-specific EHH refers to the weighted average of EHH surrounding a core SNP according to squared-allele frequencies [26]. Because site-specific EHH does not require markers to be polymorphic within the population, it can detect selective sweeps for alleles that have risen to fixation. Both *iHS* and *Rsb* were calculated using the *scan_hh* program in the R package *rehh* [44], which excludes markers with any missing data. The allele frequency-based *Tajima's D* approach [11] was used to detect balancing selection at each gene, with greater evidence from increasing values from zero. Whilst different populations have different demographic histories, it is difficult to account for this and establish multiple thresholds for the different null distributions of *Tajima’s D*. To negate any confounding effects of population expansion and difficulties in establishing significance levels, we report genes with *Tajima’s D* values in excess of one [6]. The function *neutrality*.*stats* from the R package *PopGenome* was used [45]. Parasite population structure was analysed using principal components analysis, implemented via the classical multidimensional scaling method using the function *cmdscale* in R. In addition, pairwise population *Fst* with 95% bootstrap confidence intervals was computed using the function *stamppFst* in the R package *StAMPP* [46].

## Supporting Information

S1 Fig For a sample of 1,000 SNPs (in the 86k set), there is a significant linear relationship between SNP minor allele frequency and percentage of missing genotypes (P<2x10^-16^). (PDF)

S2 Fig (A) MAF of a sample of 5,000 SNPs in each population pre-and post-imputation, using IMPUTE (recombination map: LDhat, reference panel: population-specific). No systematic increase or decrease was detected; (B) MAF of a sample of 5,000 SNPs in each population pre-and post-imputation, using Beagle. No systematic increase or decrease was detected.
(PDF)

S3 Fig Pearson’s correlation (*r)* between |*iHS*| metrics calculated from Beagle- or IMPUTE-imputed genotypes. Diagonal line indicates line of equality.
(PDF)

S4 Fig Pearson’s correlation (*r*) between *Rsb* metrics calculated from Beagle- or IMPUTE-imputed haplotypes. Malawi is used as the reference population. Diagonal line indicates line of equality. Mass of points above the line indicates that IMPUTE-derived haplotypes systematically produce larger *Rsb* values than Beagle-derived haplotypes.
(PDF)

S5 Fig Pearson’s correlation (*r*) between |*iHS*| metrics calculated from Beagle-imputed or complete-case haplotypes. Diagonal line indicates line of equality.
(PDF)

S6 Fig Pearson’s correlation (*r*) between *Rsb* metrics calculated from Beagle-imputed or complete-case haplotypes. Malawi (MLW) is used as the reference population. Diagonal line indicates line of equality.
(PDF)

S7 Fig Analysis of site-specific EHH around a core SNP within *dhfr*. With Malawi as the reference population, *Rsb* metrics indicate no signal for positive selection in *dhfr* but do indicate a signal for positive selection in neighbouring *PF3D7_0417400*. It appears that the beneficial allele is sweeping up in all 4 populations, causing attenuation of inter-population differences at *dhfr*. The longest haplotypes appear to occur in Thailand, producing the strongest signal in *PF3D7_0417400*.
(PDF)

S8 Fig Analysis of site-specific EHH around a core SNP within *dhps*. With Malawi as the reference population, *Rsb* metrics in Thai and Cambodian populations indicate no signal for positive selection in *dhps* but do indicate a strong signal for positive selection in neighbouring *PF3D7_0809800*. In contrast, *dhps* is detected by using Vietnam (VTN) as the reference population.
(PDF)

S9 Fig Principal components analysis of *P*. *falciparum* isolates using unimputed (top) and Beagle-imputed (bottom) SNP data. PC1 and PC2 refer to the first and second principal components, respectively.
(PDF)

S10 Fig Recombination maps from experimental crosses (GB4x7G8 and HB3xDD2) and LDhat inference (Gambia and Thailand). The chromosomes are ordered from 1–14 by row. The highlighted inset shows putative recombination hotspot in the middle of chromosome 3, validated in Mu et al. [47], appearing in the GB4x7G8, LDhat Gambia and Thailand maps.
(PDF)

S11 Fig SNPs ordered according to the proportion of missing genotypes (“missingness rate”) calculated prior to data filtering. A 10% cut-off based on an approximate inflection point was used.
(PDF)

S1 Table Median recombination rate per chromosome in units of cM/Mb, as estimated by LDhat, in each parasite population. Genetic maps from 2 experimental crosses were used as a benchmark to scale the compound population rate parameter (*2N*_*e*_ *r*) to obtain the LDhat genetic map distance *r* (Materials and Methods). For comparison, the recombination rate between SNP intervals in the human genome is 1–2 cM/Mb (www.hapmap.org).
(DOCX)

S2 Table Accuracy of imputed genotypes using different parameter values for *θ* and *N_e_* in IMPUTE, which are assumptions about the population mutation rate. Accuracy improves slightly by assuming a lower mutation rate for the Southeast Asian (Thailand) population but does not change for the African (Gambia) population.
(DOCX)

S3 Table Genes with SNPs in the top 1% of *|iHS|* values in each population using Beagle-imputed haplotypes, with median *|iHS|* values per gene. Only the 79 genes with 2 or more SNP hits across or within populations are shown.
(DOCX)

S4 Table Genes with SNPs in the top 1% of *|iHS|* values in each population using complete-case haplotypes, with median |*iHS*| per gene (32 genes). (DOCX)

S5 Table Genes with SNPs in the top 1% of *Rsb* values in each population using Beagle-imputed haplotypes, with Malawi as the reference population. Median *Rsb* values per gene are shown. Only the 99 genes with 2 or more SNP hits across or within populations are shown.
(DOCX)

S6 Table Genes with SNPs in the top 1% of *Rsb* values in each population using complete-case haplotypes, with Malawi as the reference population. Median *Rsb* values per gene are shown. Only the 75 genes with 2 or more SNP hits across or within populations are shown.
(DOCX)

S7 Table Genes captured by the top 1% *Rsb* metrics in Cambodia using Beagle-imputed and complete-case haplotypes, with Thailand as the reference population. Median *Rsb* values per gene are shown. Only the 103 genes with 2 or more SNP hits are shown.
(DOCX)

S8 Table Mean pairwise population *Fst* and 95% bootstrap confidence intervals using unimputed (lower triangle) and Beagle-imputed (upper triangle) genotypes. The diagonal contains the number of SNPs that are polymorphic in complete-case and post-imputation datasets.
(DOCX)

S9 Table The *P*. *falciparum* subtelomeric regions. (DOCX)
