# Correction: Imputation-Based Population Genetics Analysis of *Plasmodium falciparum* Malaria Parasites

**DOI:** 10.1371/journal.pgen.1006300

**Published:** 2016-08-31

**Authors:** The PLOS Genetics Staff

## Notice of Republication

This article was republished on July 29, 2015, to correct a duplication of S2 Fig which replaced S3 Fig. This error was introduced during the typesetting process. The publisher apologizes for the error. Please download this article again to view the correct version that includes the corrected S3 Fig. The originally published S3 Fig and corrected S3 Fig are provided here for reference.

## Supporting Information

S1 File Originally published S3 Fig. (PDF)

S2 File Republished, corrected S3 Fig. (PDF)
