# Supplementary material for: Imputation-Based Population Genetics Analysis of Plasmodium falciparum Malaria Parasites
Source: PLoS Genet. 2015 Apr 30;11(4):e1005131. doi: 10.1371/journal.pgen.1005131 (PMC4415759; doi:10.1371/journal.pgen.1005131)
Supplement: S1 Fig — (PDF) [file pgen.1005131.s001.pdf]

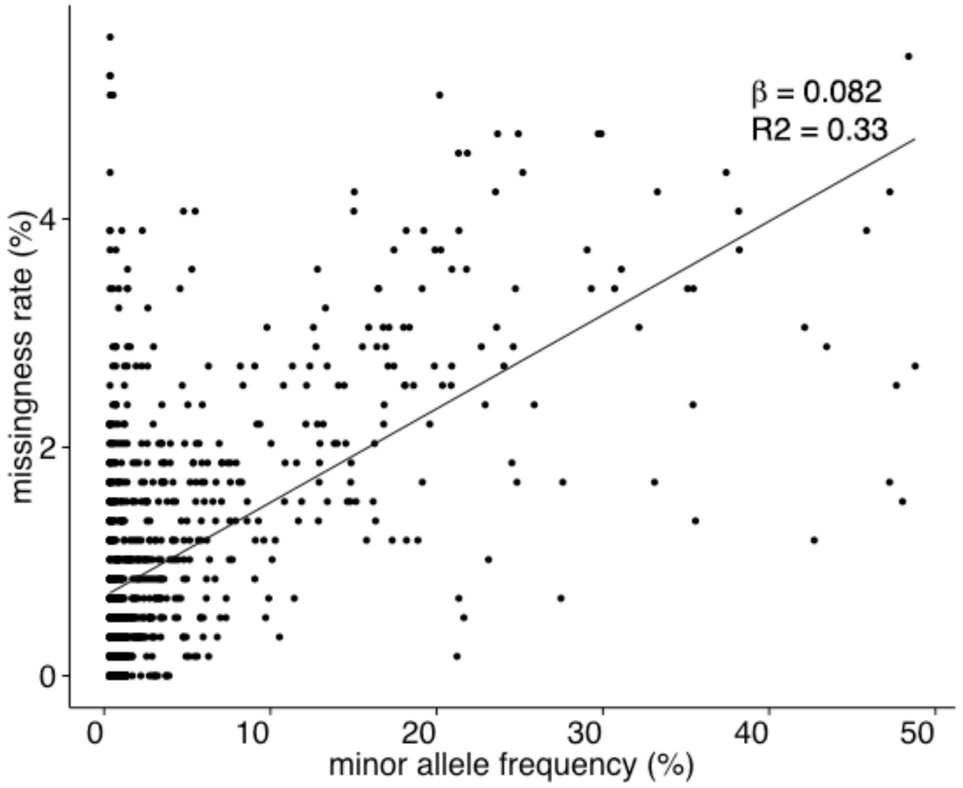

S. Figure 1. For a sample of 1,000 SNPs (in the 86k set), there is a significant linear relationship between SNP minor allele frequency and percentage of missing genotypes ($P<2x10^{-16}$).
