# Supplementary material for: Imputation-Based Population Genetics Analysis of Plasmodium falciparum Malaria Parasites
Source: PLoS Genet. 2015 Apr 30;11(4):e1005131. doi: 10.1371/journal.pgen.1005131 (PMC4415759; doi:10.1371/journal.pgen.1005131)
Supplement: S2 Fig — No systematic increase or decrease was detected; (B) MAF of a sample of 5,000 SNPs in each population pre-and post-imputation, using Beagle. No systematic increase or decrease was detected. (PDF) [file pgen.1005131.s002.pdf]

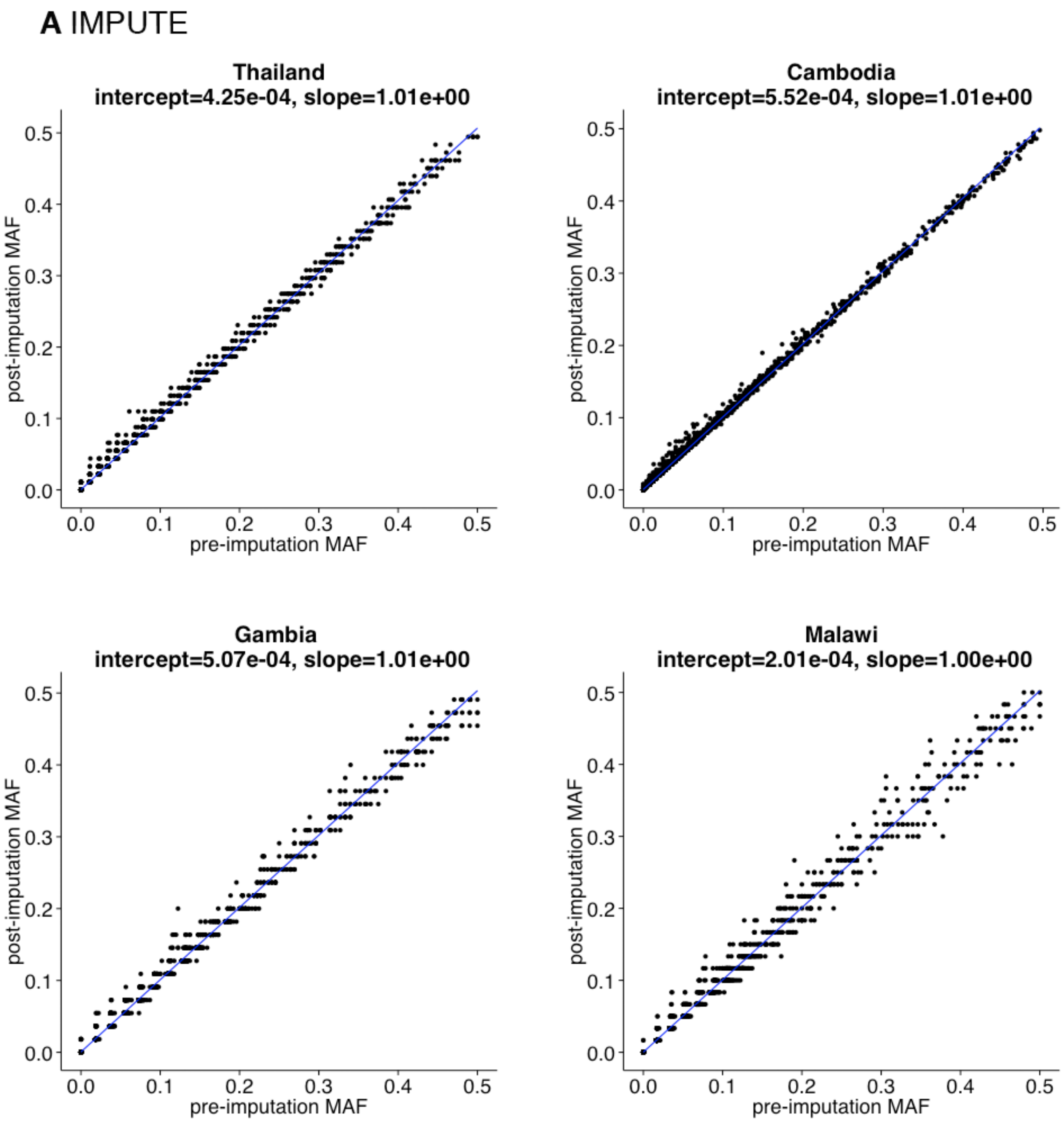

S. Figure 2A. MAF of a sample of 5,000 SNPs in each population pre-and post-imputation, using IMPUTE (recombination map: LDhat, reference panel: population-specific). No systematic increase or decrease was detected.

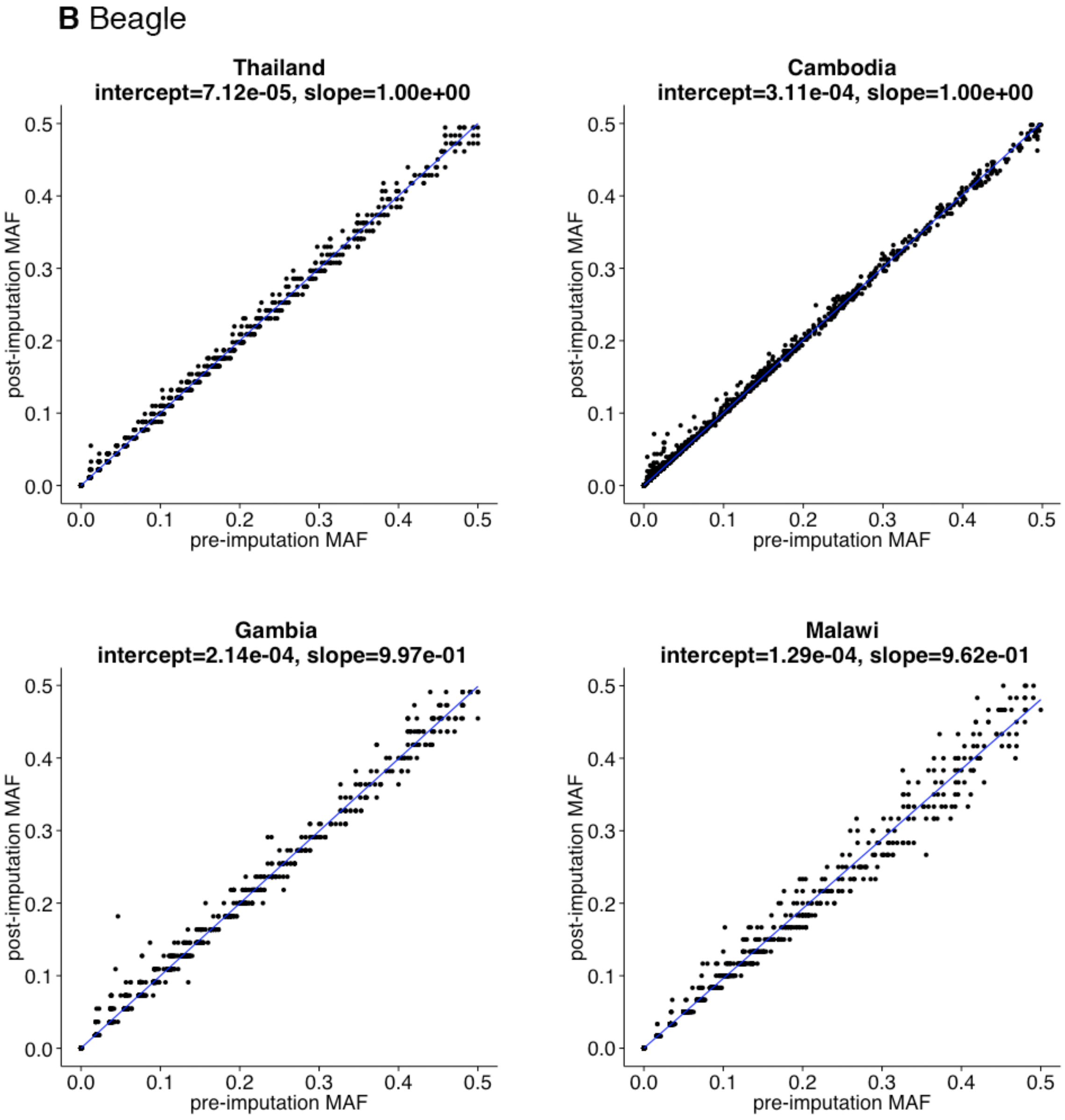

S. Figure 2B. MAF of a sample of 5,000 SNPs in each population pre-and post-imputation, using Beagle. No systematic increase or decrease was detected.
