# Supplementary material for: Imputation-Based Population Genetics Analysis of Plasmodium falciparum Malaria Parasites
Source: PLoS Genet. 2015 Apr 30;11(4):e1005131. doi: 10.1371/journal.pgen.1005131 (PMC4415759; doi:10.1371/journal.pgen.1005131)
Supplement: S3 Fig — Diagonal line indicates line of equality. (PDF) [file pgen.1005131.s003.pdf]

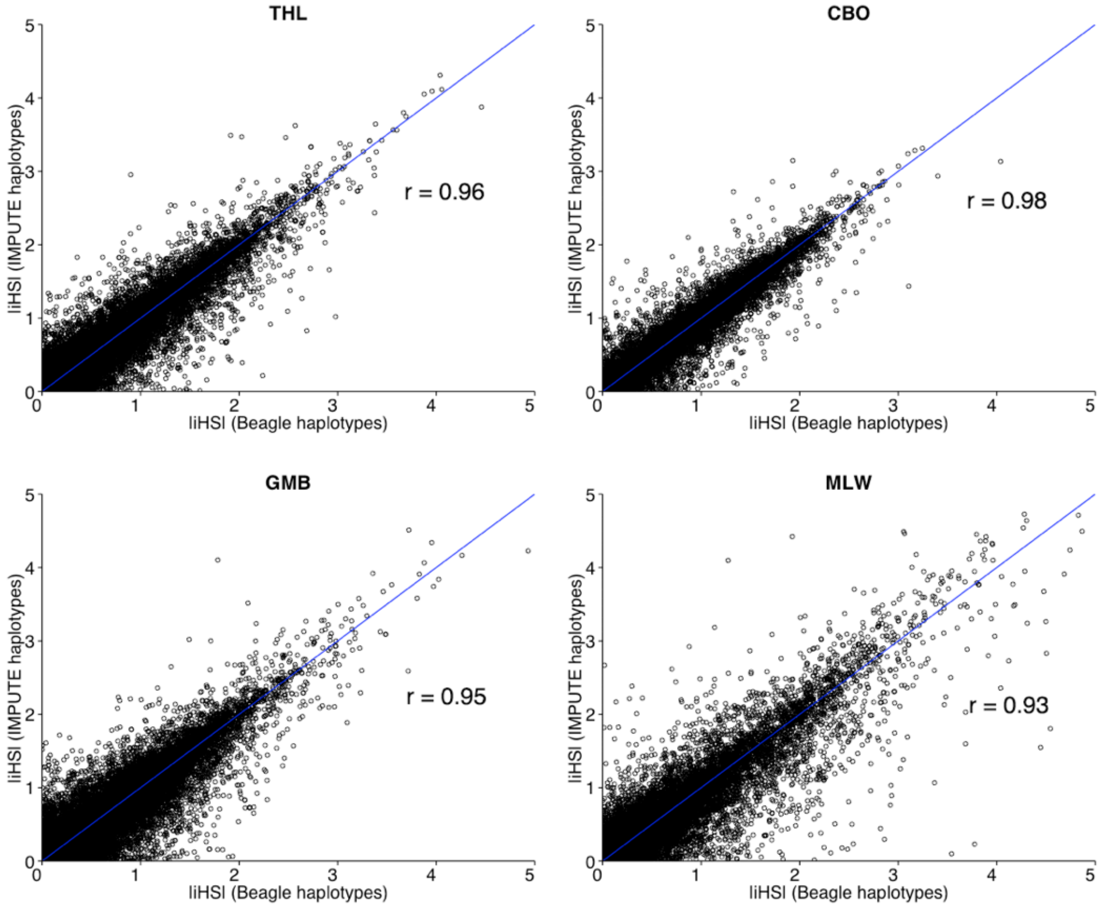

S. Figure 3. Pearson's correlation *(r)* between |*iHS*| metrics calculated from Beagle- or IMPUTE-imputed genotypes. Diagonal line indicates line of equality.
