# Supplementary material for: Imputation-Based Population Genetics Analysis of Plasmodium falciparum Malaria Parasites
Source: PLoS Genet. 2015 Apr 30;11(4):e1005131. doi: 10.1371/journal.pgen.1005131 (PMC4415759; doi:10.1371/journal.pgen.1005131)
Supplement: S4 Fig — Malawi is used as the reference population. Diagonal line indicates line of equality. Mass of points above the line indicates that IMPUTE-derived haplotypes systematically produce larger Rsb values than Beagle-derived haplotypes. (PDF) [file pgen.1005131.s004.pdf]

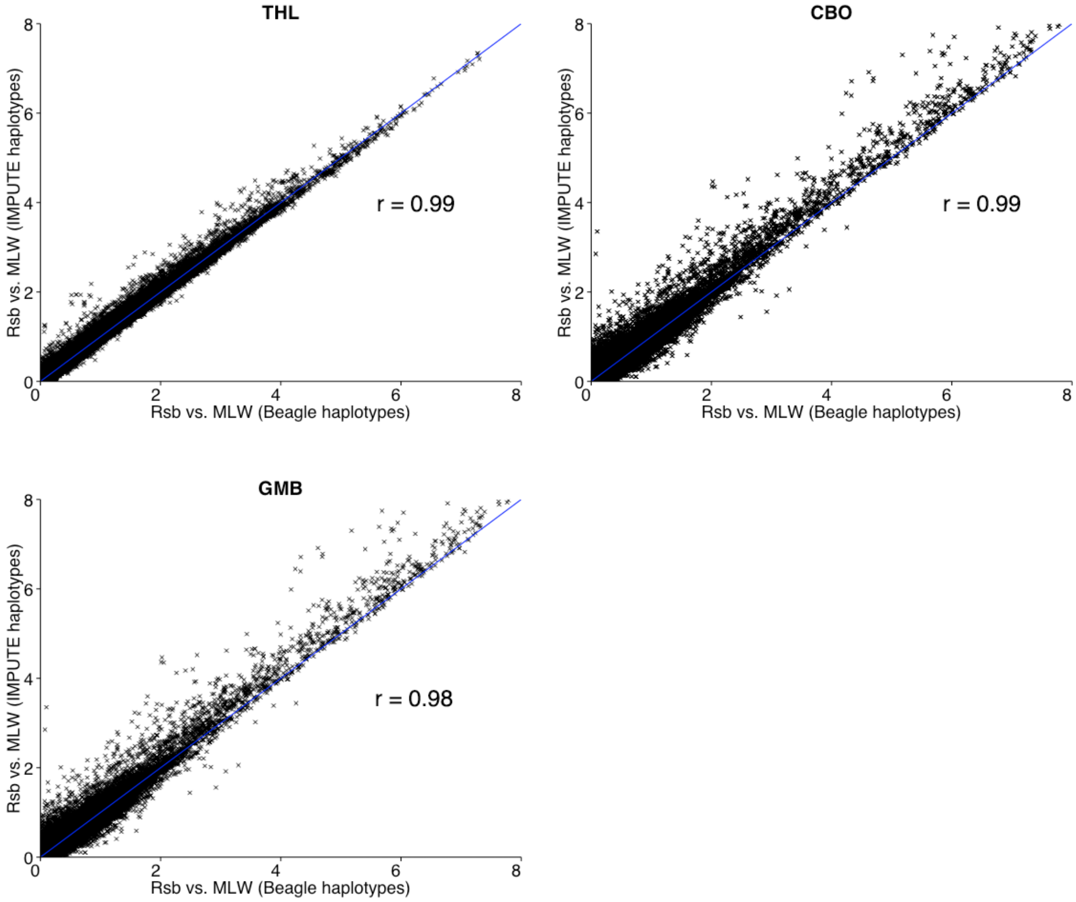

S. Figure 4. Pearson's correlation (*r*) between *Rsb* metrics calculated from Beagle- or IMPUTE-imputed haplotypes. Malawi is used as the reference population. Diagonal line indicates line of equality. Mass of points above the line indicates that IMPUTE-derived haplotypes systematically produce larger *Rsb* values than Beagle-derived haplotypes.
