# Supplementary material for: Imputation-Based Population Genetics Analysis of Plasmodium falciparum Malaria Parasites
Source: PLoS Genet. 2015 Apr 30;11(4):e1005131. doi: 10.1371/journal.pgen.1005131 (PMC4415759; doi:10.1371/journal.pgen.1005131)
Supplement: S7 Fig — With Malawi as the reference population, Rsb metrics indicate no signal for positive selection in dhfr but do indicate a signal for positive selection in neighbouring PF3D7_0417400. It appears that the beneficial allele is sweeping up in all 4 populations, causing attenuation of inter-population differences at dhfr. The longest haplotypes appear to occur in Thailand, producing the strongest signal in PF3D7_0417400. (PDF) [file pgen.1005131.s007.pdf]

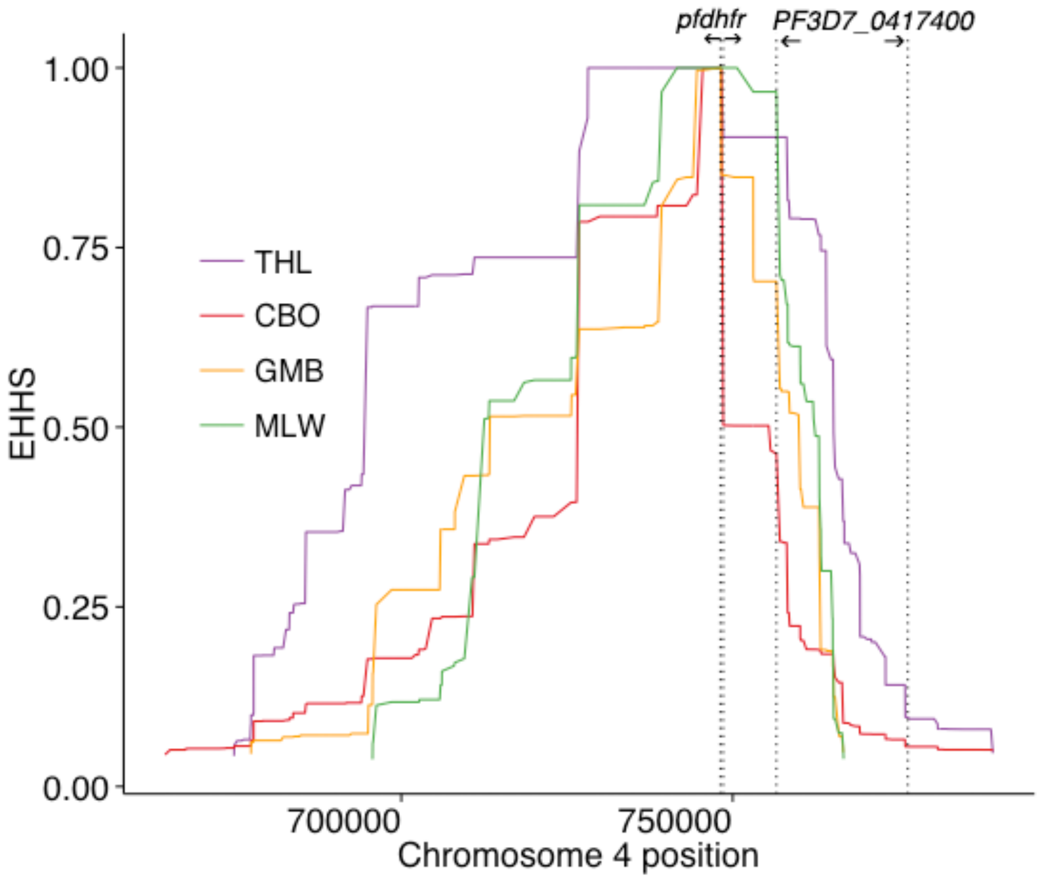

S. Figure 7. Analysis of site-specific EHH around a core SNP within *dhfr*. With Malawi as the reference population, *Rsb* metrics indicate no signal for positive selection in *dhfr* but do indicate a signal for positive selection in neighbouring *PF3D7_0417400*. It appears that the beneficial allele is sweeping up in all 4 populations, causing attenuation of inter-population differences at *dhfr*. The longest haplotypes appear to occur in Thailand, producing the strongest signal in *PF3D7_0417400*.
