# Supplementary material for: Imputation-Based Population Genetics Analysis of Plasmodium falciparum Malaria Parasites
Source: PLoS Genet. 2015 Apr 30;11(4):e1005131. doi: 10.1371/journal.pgen.1005131 (PMC4415759; doi:10.1371/journal.pgen.1005131)
Supplement: S8 Fig — With Malawi as the reference population, Rsb metrics in Thai and Cambodian populations indicate no signal for positive selection in dhps but do indicate a strong signal for positive selection in neighbouring PF3D7_0809800. In contrast, dhps is detected by using Vietnam (VTN) as the reference population. (PDF) [file pgen.1005131.s008.pdf]

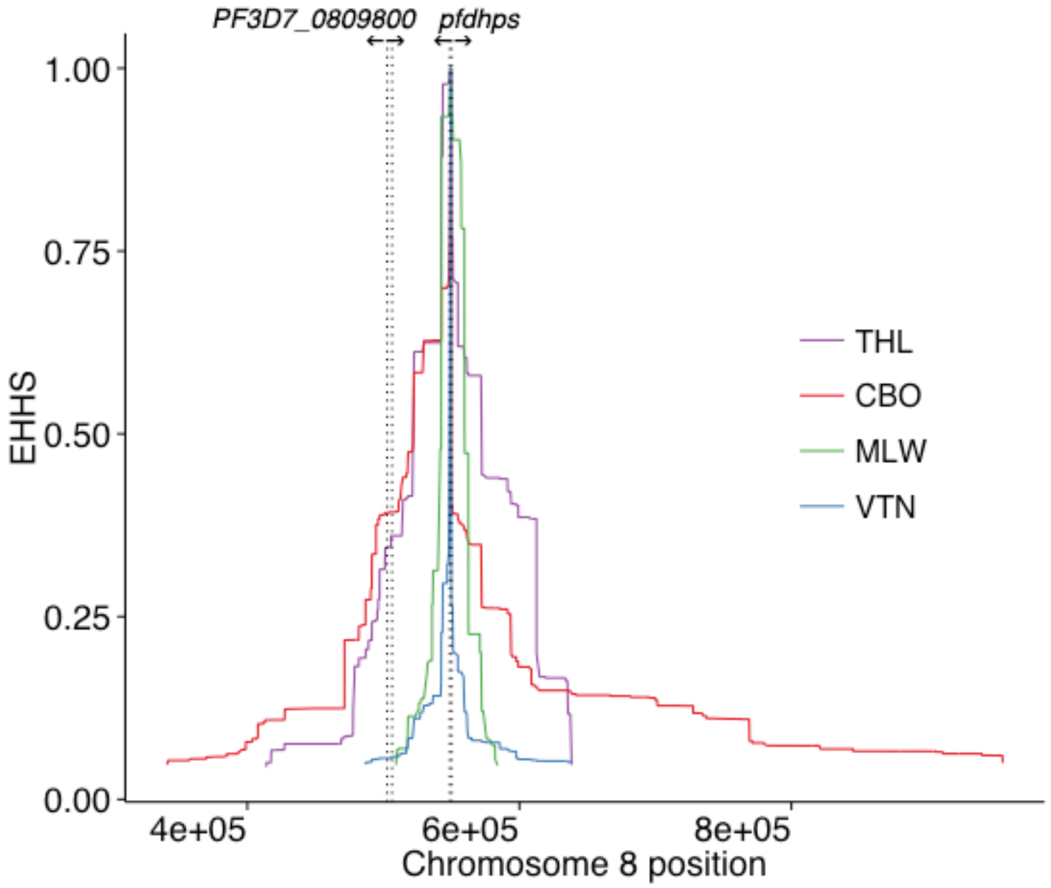

S. Figure 8. Analysis of site-specific EHH around a core SNP within *dhps*. With Malawi as the reference population, *Rsb* metrics in Thai and Cambodian populations indicate no signal for positive selection in *dhps* but do indicate a strong signal for positive selection in neighbouring *PF3D7_0809800*. In contrast, *dhps* is detected by using Vietnam (VTN) as the reference population.
