# Supplementary material for: Imputation-Based Population Genetics Analysis of Plasmodium falciparum Malaria Parasites
Source: PLoS Genet. 2015 Apr 30;11(4):e1005131. doi: 10.1371/journal.pgen.1005131 (PMC4415759; doi:10.1371/journal.pgen.1005131)
Supplement: S9 Fig — PC1 and PC2 refer to the first and second principal components, respectively. (PDF) [file pgen.1005131.s009.pdf]

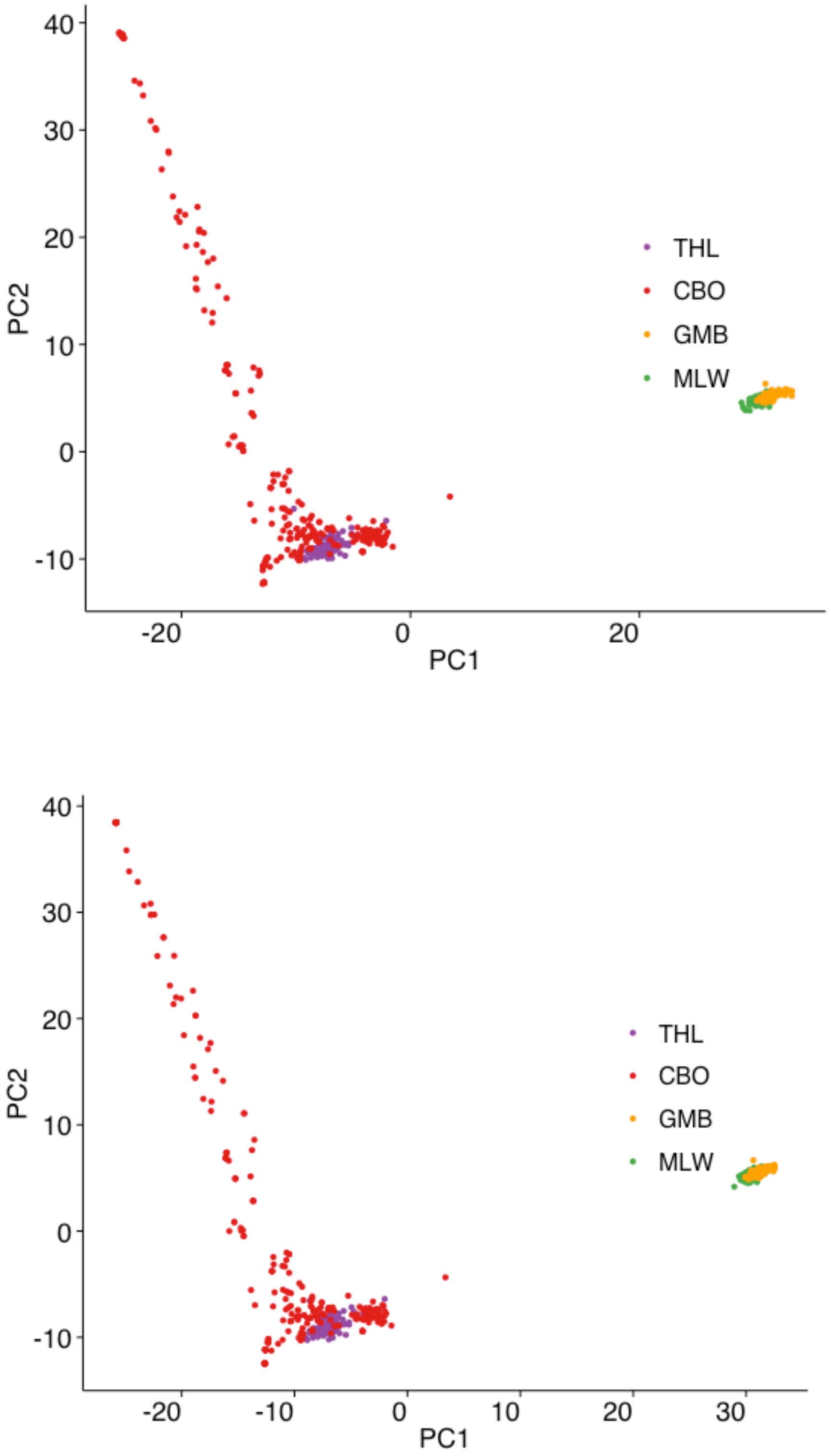

S. Figure 9. Principal components analysis of *P. falciparum* isolates using unimputed (top) and Beagle-imputed (bottom) SNP data. PC1 and PC2 refer to the first and second principal components, respectively.
