# Supplementary material for: Imputation-Based Population Genetics Analysis of Plasmodium falciparum Malaria Parasites
Source: PLoS Genet. 2015 Apr 30;11(4):e1005131. doi: 10.1371/journal.pgen.1005131 (PMC4415759; doi:10.1371/journal.pgen.1005131)
Supplement: S10 Fig — The chromosomes are ordered from 1–14 by row. The highlighted inset shows putative recombination hotspot in the middle of chromosome 3, validated in Mu et al. [47], appearing in the GB4x7G8, LDhat Gambia and Thailand maps. (PDF) [file pgen.1005131.s010.pdf]

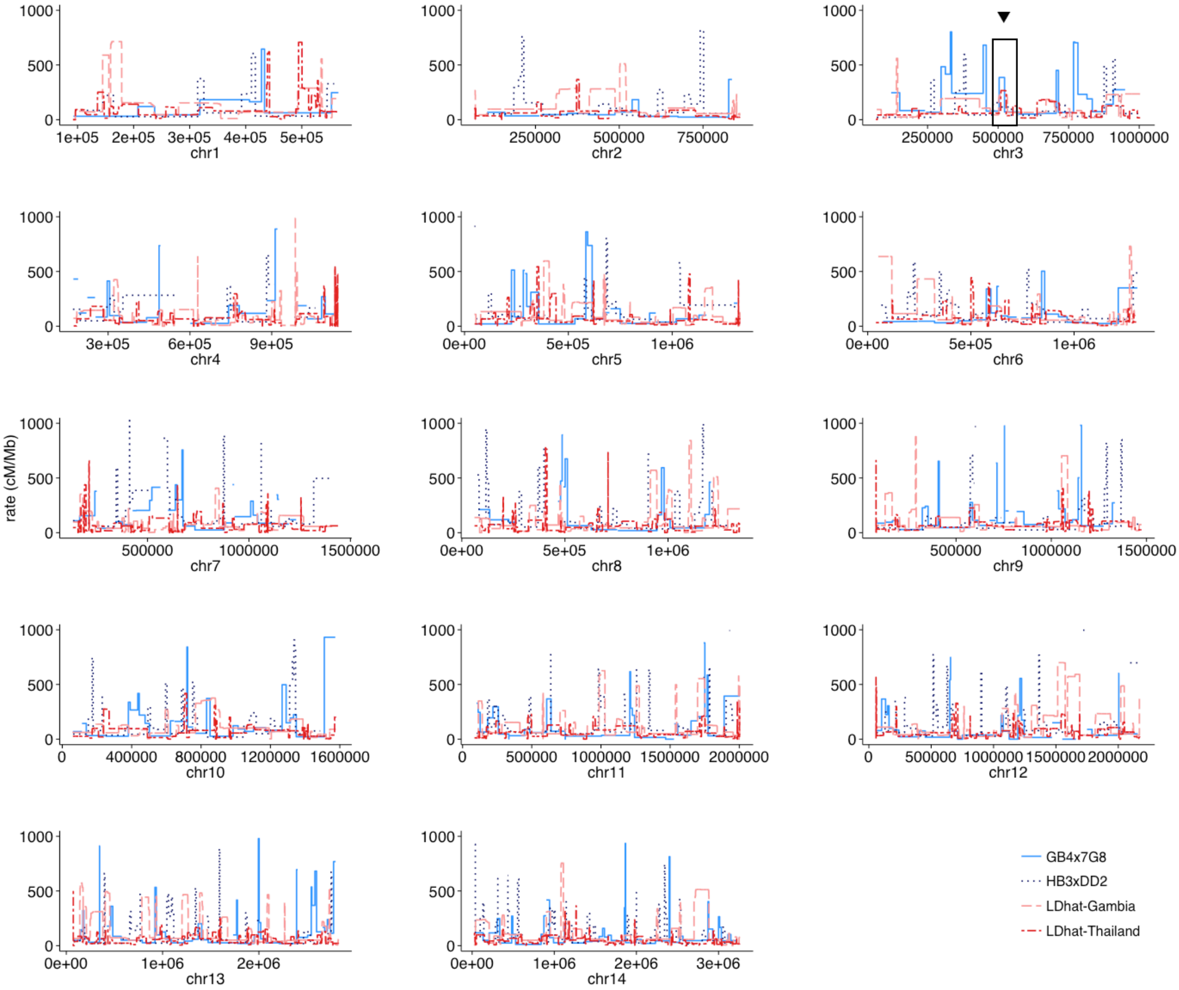

S. Figure 10. Recombination maps from experimental crosses (GB4x7G8 and HB3xDD2) and LDhat inference (Gambia and Thailand). The chromosomes are ordered from 1-14 by row. The highlighted inset shows putative recombination hotspot in the middle of chromosome 3, validated in Mu (47), appearing in the GB4x7G8, LDhat Gambia and Thailand maps.
