# Supplementary material for: Imputation-Based Population Genetics Analysis of Plasmodium falciparum Malaria Parasites
Source: PLoS Genet. 2015 Apr 30;11(4):e1005131. doi: 10.1371/journal.pgen.1005131 (PMC4415759; doi:10.1371/journal.pgen.1005131)
Supplement: S11 Fig — A 10% cut-off based on an approximate inflection point was used. (PDF) [file pgen.1005131.s011.pdf]

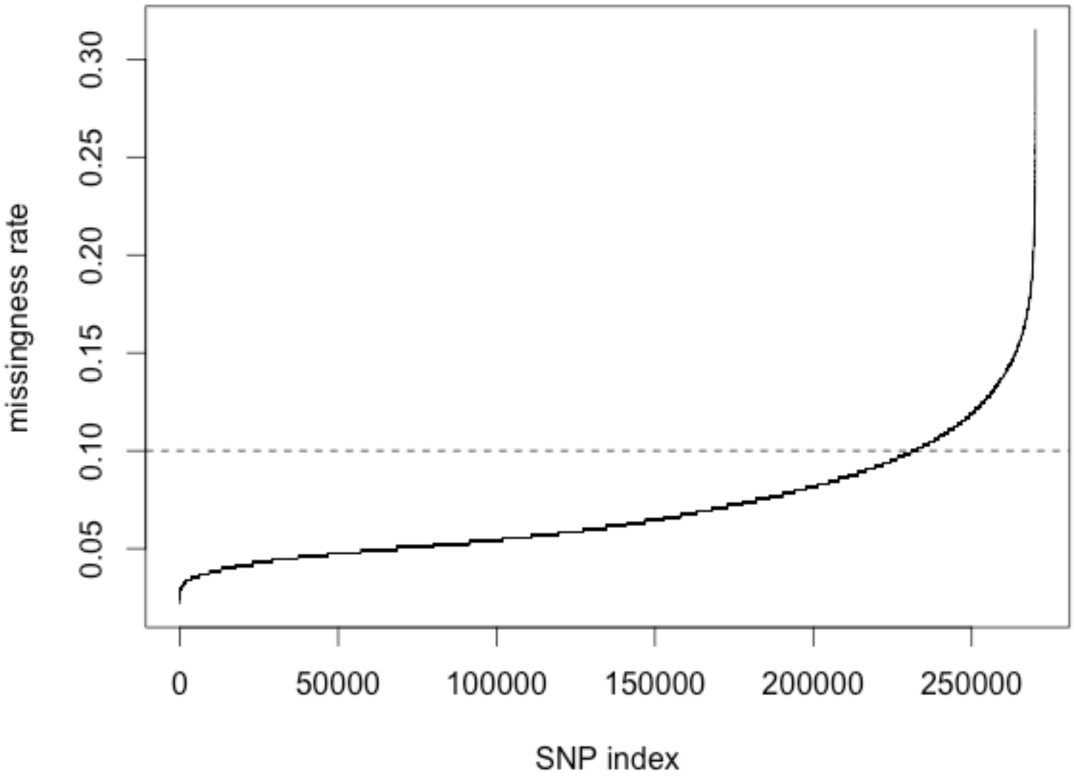

S. Figure 11. SNPs ordered according to the proportion of missing genotypes ("missingness rate") calculated prior to data filtering. A 10% cut-off based on an approximate inflection point was used.
