# Supplementary material for: Imputation-Based Population Genetics Analysis of Plasmodium falciparum Malaria Parasites
Source: PLoS Genet. 2015 Apr 30;11(4):e1005131. doi: 10.1371/journal.pgen.1005131 (PMC4415759; doi:10.1371/journal.pgen.1005131)
Supplement: S1 Table — Genetic maps from 2 experimental crosses were used as a benchmark to scale the compound population rate parameter (2N e r) to obtain the LDhat genetic map distance r (Materials and Methods). For comparison, the recombination rate between SNP intervals in the human genome is 1–2 cM/Mb (www.hapmap.org). (DOCX) [file pgen.1005131.s012.docx]

**S1 Table.** Median recombination rate per chromosome in units of cM/Mb, as estimated by LDhat, in each parasite population. Genetic maps from 2 experimental crosses were used as a benchmark to scale the compound population rate parameter (*2N_e_r*) to obtain the LDhat genetic map distance *r* (Materials and Methods). For comparison, the recombination rate between SNP intervals in the human genome is 1-2 cM/Mb (www.hapmap.org).

| **Chr** | **Thailand**  **(cM/Mb)** | **Cambodia**  **(cM/Mb)** | **Gambia**  **(cM/Mb)** | **Malawi**  **(cM/Mb)** |
| --- | --- | --- | --- | --- |
| 1 | 73.0 | 63.2 | 156.5 | 570.0 |
| 2 | 31.6 | 37.5 | 86.3 | 500.3 |
| 3 | 44.3 | 35.7 | 75.9 | 517.6 |
| 4 | 26.8 | 47.7 | 57.5 | 531.7 |
| 5 | 35.3 | 32.4 | 69.5 | 521.3 |
| 6 | 41.1 | 30.1 | 46.4 | 392.9 |
| 7 | 19.3 | 25.4 | 59.9 | 493.7 |
| 8 | 34.8 | 36.6 | 53.1 | 479.5 |
| 9 | 39.6 | 33.1 | 42.8 | 398.1 |
| 10 | 39.8 | 36.9 | 55.4 | 411.1 |
| 11 | 32.2 | 28.3 | 46.1 | 391.7 |
| 12 | 34.1 | 31.9 | 59.6 | 389.0 |
| 13 | 25.9 | 29.6 | 49.5 | 458.5 |
| 14 | 29.2 | 25.3 | 41.5 | 458.4 |
