# Supplementary material for: Imputation-Based Population Genetics Analysis of Plasmodium falciparum Malaria Parasites
Source: PLoS Genet. 2015 Apr 30;11(4):e1005131. doi: 10.1371/journal.pgen.1005131 (PMC4415759; doi:10.1371/journal.pgen.1005131)
Supplement: S2 Table — Accuracy improves slightly by assuming a lower mutation rate for the Southeast Asian (Thailand) population but does not change for the African (Gambia) population. (DOCX) [file pgen.1005131.s013.docx]

**S2 Table.** Accuracy of imputed genotypes using different parameter values for *θ* and *N_e_* in IMPUTE, which are assumptions about the population mutation rate. Accuracy improves slightly by assuming a lower mutation rate for the Southeast Asian (Thailand) population but does not change for the African (Gambia) population.

| **Population** | **n** | **% of 86k SNPs that are polymorphic** | **% SNPs with any missing data** | **Imputation method (parametervalues)** | **Refer. haplo.** | **Mean *r^2^* by SNP** | **Mean minor allele**  **concord.**  **(%)** |
| --- | --- | --- | --- | --- | --- | --- | --- |
| **Thailand** | 91 | 42% | 34% | IMPUTE2 | 90 THL | 0.56 | 46.1 |
| **(THL)** |  |  |  | (LDhat: *θ*=0.01, *N_e_*=30,000) |  |  |  |
|  |  |  |  | IMPUTE2 | 90 THL | 0.62 | 53.2 |
|  |  |  |  | (LDhat: *θ*=0.001, *N_e_*=30,000) |  |  |  |
|  |  |  |  | IMPUTE2 | 90 THL | 0.64 | 62.5 |
|  |  |  |  | (LDhat:  *θ*=0.01, *N_e_*=10,000) |  |  |  |
| **Gambia** | 55 | 38% | 38% | IMPUTE2 | 54 GMB | 0.99 | 95.4 |
| **(GMB)** |  |  |  | (LDhat: *θ*=0.01, *N_e_*=30,000) |  |  |  |
|  |  |  |  | IMPUTE2 | 54 GMB | 0.99 | 95.3 |
|  |  |  |  | (LDhat: *θ*=0.001, *N_e_*=30,000) |  |  |  |
|  |  |  |  | IMPUTE2 | 54 GMB | 0.99 | 95.4 |
|  |  |  |  | (LDhat: *θ*=0.01, *N_e_*=10,000) |  |  |  |
