# Supplementary material for: Imputation-Based Population Genetics Analysis of Plasmodium falciparum Malaria Parasites
Source: PLoS Genet. 2015 Apr 30;11(4):e1005131. doi: 10.1371/journal.pgen.1005131 (PMC4415759; doi:10.1371/journal.pgen.1005131)
Supplement: S3 Table — Only the 79 genes with 2 or more SNP hits across or within populations are shown. (DOCX) [file pgen.1005131.s014.docx]

**S3 Table.** Genes with SNPs in the top 1% of *|iHS|* values in each population using Beagle-imputed haplotypes, with median |*iHS|* values per gene. Only the 79 genes with 2 or more SNP hits across or within populations are shown.

| **Chr** | **Gene**  **ID** | **Gene**  **name** | **Thailand (iHS)** | **Cambodia**  **(iHS)** | **Gambia**  **(iHS)** | **Malawi (iHS)** | **Total SNPs** |
| --- | --- | --- | --- | --- | --- | --- | --- |
| 1 | *PF3D7_0113800* |  | 2.71 | 2.77 | 2.49 | 3.91 | 16 |
| 1 | *PF3D7_0113600* | *SURFIN1.2* | 3.05 | 2.40 | - | - | 10 |
| 2 | *PF3D7_0220000* | *LSA3* | - | - | 2.63 | 3.81 | 3 |
| 2 | *PF3D7_0220800* | *CLAG2* | 2.91 | 2.53 | 2.78 | - | 5 |
| 2 | *PF3D7_0221000* |  | 2.87 | - | - | - | 3 |
| 3 | *PF3D7_0322700* |  | 2.88 | - | 2.57 | - | 2 |
| 3 | *PF3D7_0304700* |  | 2.57 | 2.29 | - | - | 2 |
| 3 | *PF3D7_0308200* |  | - | 2.58 | - | - | 2 |
| 4 | *PF3D7_0411900* |  | - | - | 2.79 | 3.18 | 2 |
| 4 | *PF3D7_0414000* |  | - | - | 2.49 | - | 2 |
| 4 | *PF3D7_0417400* |  | 2.76 | - | 2.80 | 3.00 | 8 |
| 4 | *PF3D7_0419900* |  | - | 2.31 | 2.60 | - | 2 |
| 4 | *PF3D7_0420000* |  | - | - | 2.45 | 3.64 | 5 |
| 4 | *PF3D7_0421700* |  | - | - | 2.76 | 2.98 | 6 |
| 4 | *PF3D7_0411800* |  | 2.73 | - | - | - | 2 |
| 4 | *PF3D7_0425000* |  | 2.73 | 2.42 | - | - | 4 |
| 5 | *PF3D7_0526600* |  | - | 2.58 | 2.80 | - | 5 |
| 5 | *PF3D7_0532800* |  | 3.08 | - | - | - | 2 |
| 5 | *PF3D7_0508900* |  | - | 2.24 | - | 3.33 | 2 |
| 5 | *PF3D7_0524300* |  | - | - | - | 3.34 | 3 |
| 7 | *PF3D7_0709100* | *CG1* | - | - | 2.52 | 3.95 | 13 |
| 7 | *PF3D7_0709300* | *CG2* | - | 2.34 | 2.57 | - | 10 |
| 7 | *PF3D7_0709600* |  | - | 2.54 | 2.41 | - | 2 |
| 7 | *PF3D7_0710000* |  | - | 2.39 | 2.70 | - | 10 |
| 7 | *PF3D7_0710200* |  | - | - | 2.54 | 2.99 | 7 |
| 7 | *PF3D7_0713600* |  | 2.86 | - | - | - | 6 |
| 7 | *PF3D7_0709400* | *CG7* | - | 2.57 | - | 3.01 | 2 |
| 8 | *PF3D7_0808300* |  | - | - | 2.69 | - | 2 |
| 8 | *PF3D7_0809200* | *pfa55-14* | - | - | 2.50 | 3.21 | 4 |
| 8 | *PF3D7_0809400* |  | - | - | 2.61 | - | 2 |
| 8 | *PF3D7_0809600* |  | 3.22 | 2.43 | 2.41 | - | 13 |
| 8 | *PF3D7_0826000* |  | - | - | 2.37 | 3.18 | 3 |
| 9 | *PF3D7_0901700* |  | 2.59 | - | 2.96 | - | 2 |
| 9 | *PF3D7_0903400* | *DEAD/DEAH* | 2.71 | 2.53 | 2.63 | 3.21 | 8 |
| 9 | *PF3D7_0913900* |  | - | 2.39 | 2.90 | - | 2 |
| 9 | *PF3D7_0916400* |  | - | - | 2.60 | 3.23 | 3 |
| 9 | *PF3D7_0919900* |  | - | - | 2.48 | - | 3 |
| 9 | *PF3D7_0903300* |  | 2.73 | - | - | - | 4 |
| 9 | *PF3D7_0903500* |  | 2.57 | 2.28 | - | 3.18 | 3 |
| 9 | *PF3D7_0914100* |  | 2.81 | - | - | - | 2 |
| 9 | *PF3D7_0914000* |  | - | 2.30 | - | 3.69 | 4 |
| 10 | *PF3D7_1004600* |  | 3.04 | 2.25 | 2.51 | 3.15 | 4 |
| 10 | *PF3D7_1033100* | *AdoMetDC* | - | - | 2.81 | - | 2 |
| 10 | *PF3D7_1034900* |  | - | 2.32 | 2.61 | 3.81 | 5 |
| 10 | *PF3D7_1035000* |  | 3.09 | 2.47 | 2.53 | 4.46 | 6 |
| 10 | *PF3D7_1035100* |  | 2.81 | 2.38 | 2.55 | 4.29 | 21 |
| 10 | *PF3D7_1039000* | *FIKK10.2* | - | - | 2.79 | 3.24 | 4 |
| 10 | *PF3D7_1035300* | *GLURP* | - | 2.45 | - | 3.23 | 2 |
| 11 | *PF3D7_1133400* | *AMA1* | 2.84 | 2.52 | 3.29 | 3.20 | 20 |
| 11 | *PF3D7_1149200* | *RIESA* | 3.31 | - | - | - | 2 |
| 11 | *PF3D7_1140900* |  | - | 2.29 | - | 3.70 | 2 |
| 11 | *PF3D7_1149600* |  | - | 2.82 | - | - | 3 |
| 12 | *PF3D7_1201400* |  | 3.30 | 2.67 | - | 4.15 | 6 |
| 12 | *PF3D7_1208100* |  | 2.60 | 2.60 | - | - | 3 |
| 12 | *PF3D7_1220300* |  | 3.24 | - | - | 3.15 | 2 |
| 12 | *PF3D7_1238500* |  | 2.70 | - | - | - | 2 |
| 12 | *PF3D7_1239800* |  | 2.69 | 2.28 | - | - | 6 |
| 13 | *PF3D7_1301800* | *SURFIN13.1* | 2.70 | 2.42 | 3.21 | 3.02 | 10 |
| 13 | *PF3D7_1335900* | *TRAP* | 3.38 | - | 2.86 | 3.72 | 17 |
| 13 | *PF3D7_1352900* |  | - | - | 2.98 | 3.25 | 6 |
| 13 | *PF3D7_1368800* |  | - | - | 2.46 | - | 3 |
| 13 | *PF3D7_1371600* | *EBL1* | - | - | 2.50 | - | 2 |
| 13 | *PF3D7_1302900* |  | 2.78 | 2.57 | - | - | 2 |
| 13 | *PF3D7_1306500* |  | 2.79 | - | - | - | 4 |
| 13 | *PF3D7_1339700* |  | 2.81 | - | - | 3.23 | 2 |
| 13 | *PF3D7_1349500* |  | 2.63 | 2.55 | - | - | 2 |
| 13 | *PF3D7_1324300* |  | - | - | - | 3.03 | 2 |
| 14 | *PF3D7_1438400* | *MCA2* | - | - | 2.50 | 3.24 | 2 |
| 14 | *PF3D7_1448500* |  | - | - | 2.48 | 3.31 | 3 |
| 14 | *PF3D7_1475800* |  | 3.11 | 2.84 | 3.40 | - | 13 |
| 14 | *PF3D7_1475900* |  | - | - | 2.99 | 4.47 | 8 |
| 14 | *PF3D7_1428500* |  | 2.77 | - | - | - | 2 |
| 14 | *PF3D7_1453600* |  | 2.76 | - | - | - | 2 |
| 14 | *PF3D7_1474000* |  | 2.68 | 3.13 | - | - | 2 |
| 14 | *PF3D7_1429900* |  | - | 2.36 | - | 3.28 | 2 |
| 14 | *PF3D7_1401200* |  | - | - | - | 2.98 | 2 |
| 14 | *PF3D7_1467600* |  | - | - | - | 3.27 | 3 |
