# Supplementary material for: Imputation-Based Population Genetics Analysis of Plasmodium falciparum Malaria Parasites
Source: PLoS Genet. 2015 Apr 30;11(4):e1005131. doi: 10.1371/journal.pgen.1005131 (PMC4415759; doi:10.1371/journal.pgen.1005131)
Supplement: S4 Table — (DOCX) [file pgen.1005131.s015.docx]

**S4 Table.** Genes with SNPs in the top 1% of *|iHS|* values in each population using complete-case haplotypes, with median *|iHS|* per gene (32 genes).

| **Chr** | **Gene**  **ID** | **Gene name** | **Thailand (iHS)** | **Cambodia (iHS)** | **Gambia (iHS)** | **Malawi (iHS)** | **Total SNPs** |
| --- | --- | --- | --- | --- | --- | --- | --- |
| 1 | *PF3D7_0103600* |  | 6.33 | - | - | - | 1 |
| 1 | *PF3D7_0104100* |  | 4.78 | - | - | - | 1 |
| 2 | *PF3D7_0221300* |  | 2.98 | - | - | - | 1 |
| 3 | *PF3D7_0318500* |  | - | - | 3.57 | - | 1 |
| 3 | *PF3D7_0322500* |  | 6.84 | - | - | - | 1 |
| 3 | *PF3D7_0307900* |  | - | - | - | 4.84 | 1 |
| 4 | *PF3D7_0405300* | *LISP2* | - | - | 3.27 | - | 4 |
| 4 | *PF3D7_0419900* |  | 5.01 | - | - | - | 1 |
| 5 | *PF3D7_0522600* |  | 2.94 | - | - | - | 1 |
| 5 | *PF3D7_0504800* |  | - | - | - | 3.21 | 1 |
| 7 | *PF3D7_0714100* |  | 3.18 | - | - | - | 1 |
| 7 | *PF3D7_0711400* |  | - | - | - | 3.66 | 2 |
| 9 | *PF3D7_0903300* |  | 3.27 | - | - | - | 1 |
| 9 | *PF3D7_0932100* | *MAM3* | - | - | - | 3.84 | 1 |
| 11 | *PF3D7_1133400* | *AMA1* | - | - | 3.53 | - | 1 |
| 11 | *PF3D7_1113000* |  | - | - | - | 3.17 | 1 |
| 12 | *PF3D7_1218200* |  | - | - | 3.20 | - | 1 |
| 12 | *PF3D7_1205100* | *SEPSECS* | 2.97 | - | - | - | 1 |
| 12 | *PF3D7_1208100* |  | - | 3.48 | - | - | 1 |
| 13 | *PF3D7_1324300* |  | - | - | 3.67 | - | 1 |
| 13 | *PF3D7_1335100* | *MSP7* | 4.30 | - | - | - | 1 |
| 13 | *PF3D7_1322300* |  | - | 3.23 | - | - | 1 |
| 13 | *PF3D7_1352900* |  | - | 3.03 | - | - | 1 |
| 13 | *PF3D7_1329800* |  | - | - | - | 6.70 | 1 |
| 14 | *PF3D7_1440400* |  | - | - | 3.83 | - | 1 |
| 14 | *PF3D7_1442700* |  | - | - | 3.25 | - | 1 |
| 14 | *PF3D7_1458800* |  | - | - | 4.28 | - | 1 |
| 14 | *PF3D7_1469400* | *NT3* | - | - | 2.90 | - | 1 |
| 14 | *PF3D7_1408200* | *ApiAP2* | 3.19 | - | - | - | 2 |
| 14 | *PF3D7_1431300* |  | 5.85 | - | - | - | 1 |
| 14 | *PF3D7_1446500* |  | 3.28 | - | - | - | 1 |
| 14 | *PF3D7_1406300* | *GDPD* | - | 3.01 | - | - | 1 |
