# Supplementary material for: Imputation-Based Population Genetics Analysis of Plasmodium falciparum Malaria Parasites
Source: PLoS Genet. 2015 Apr 30;11(4):e1005131. doi: 10.1371/journal.pgen.1005131 (PMC4415759; doi:10.1371/journal.pgen.1005131)
Supplement: S5 Table — Median Rsb values per gene are shown. Only the 99 genes with 2 or more SNP hits across or within populations are shown. (DOCX) [file pgen.1005131.s016.docx]

**S5 Table.** Genes with SNPs in the top 1% of *Rsb* values in each population using Beagle-imputed haplotypes, with Malawi as the reference population. Median *Rsb* values per gene are shown. Only the 99 genes with 2 or more SNP hits across or within populations are shown.

| **Chr** | **Gene ID** | **Gene**  **name** | **Thailand (Rsb)** | **Cambodia (Rsb)** | **Gambia (Rsb)** | **Total SNPs** |
| --- | --- | --- | --- | --- | --- | --- |
| 1 | *PF3D7_0103600* |  | - | 3.43 | - | 2 |
| 1 | *PF3D7_0104100* |  | - | 3.41 | - | 2 |
| 1 | *PF3D7_0113600* | *SURFIN1.2* | - | 3.98 | - | 17 |
| 1 | *PF3D7_0113800* |  | - | 4.43 | - | 59 |
| 3 | *PF3D7_0321300* |  | 3.66 | 3.42 | - | 8 |
| 3 | *PF3D7_0304600* | *CSP* | - | 3.48 | - | 3 |
| 4 | *PF3D7_0412300* |  | 3.77 | 3.57 | 4.12 | 13 |
| 4 | *PF3D7_0413900* | *USP13* | - | - | 3.44 | 3 |
| 4 | *PF3D7_0414000* |  | 4.04 | - | 3.74 | 39 |
| 4 | *PF3D7_0414100* |  | 3.79 | - | 3.98 | 29 |
| 4 | *PF3D7_0414500* |  | - | - | 3.52 | 2 |
| 4 | *PF3D7_0414600* |  | - | - | 3.56 | 2 |
| 4 | *PF3D7_0415200* |  | - | - | 4.01 | 8 |
| 4 | *PF3D7_0415300* | *CRK3* | - | - | 3.77 | 4 |
| 4 | *PF3D7_0415700* |  | 3.83 | - | 4.46 | 4 |
| 4 | *PF3D7_0415800* |  | 4.54 | 3.41 | 4.04 | 28 |
| 4 | *PF3D7_0417400* |  | 4.03 | 3.38 | - | 16 |
| 4 | *PF3D7_0421700* |  | 3.59 | 3.72 | - | 8 |
| 5 | *PF3D7_0511400* |  | 4.17 | 3.64 | 3.06 | 11 |
| 5 | *PF3D7_0511300* |  | 3.78 | 3.64 | - | 21 |
| 5 | *PF3D7_0526600* |  | 4.22 | 4.21 | - | 14 |
| 5 | *PF3D7_0529000* |  | 3.85 | 3.88 | - | 3 |
| 5 | *PF3D7_0508900* |  | - | 3.45 | - | 2 |
| 7 | *PF3D7_0708200* |  | 4.84 | 4.50 | 5.40 | 33 |
| 7 | *PF3D7_0708300* |  | 5.16 | 4.56 | 6.20 | 12 |
| 7 | *PF3D7_0708400* | *HSP90* | 5.60 | 4.91 | 8.21 | 48 |
| 7 | *PF3D7_0708500* | *HSP86* | 3.93 | 3.51 | 4.83 | 30 |
| 7 | *PF3D7_0708700* | *Cg8* | 4.09 | 3.42 | 4.89 | 4 |
| 7 | *PF3D7_0708800* | *HSP110c* | - | - | 3.72 | 4 |
| 7 | *PF3D7_0708900* | *Cg3* | - | - | 3.50 | 2 |
| 7 | *PF3D7_0709000* | *CRT* | 3.55 | 3.40 | 4.11 | 40 |
| 7 | *PF3D7_0709050* |  | 3.83 | - | 4.60 | 4 |
| 7 | *PF3D7_0709100* | *Cg1* | 5.98 | 4.87 | 8.91 | 111 |
| 7 | *PF3D7_0709200* | *GLP3* | 4.59 | 4.22 | 6.84 | 18 |
| 7 | *PF3D7_0709300* | *Cg2* | 5.14 | 4.61 | 8.74 | 196 |
| 7 | *PF3D7_0709400* | *Cg7* | 3.87 | 3.56 | 5.88 | 50 |
| 7 | *PF3D7_0709500* |  | - | - | 4.87 | 3 |
| 7 | *PF3D7_0709600* |  | 3.90 | 3.64 | 6.03 | 111 |
| 7 | *PF3D7_0709700* |  | - | - | 4.27 | 6 |
| 7 | *PF3D7_0709800* |  | - | - | 3.44 | 2 |
| 7 | *PF3D7_0709900* |  | - | - | 3.40 | 4 |
| 7 | *PF3D7_0710000* |  | 4.61 | 4.07 | 5.01 | 184 |
| 7 | *PF3D7_0710100* |  | - | 3.57 | 3.52 | 3 |
| 7 | *PF3D7_0710200* |  | 3.99 | 4.35 | 4.47 | 152 |
| 7 | *PF3D7_0711500* |  | 3.55 | 3.66 | 3.50 | 12 |
| 7 | *PF3D7_0713900* |  | - | 3.74 | 3.10 | 6 |
| 7 | *PF3D7_0704600* |  | 3.89 | 4.00 | - | 29 |
| 7 | *PF3D7_0707300* | *RAMA* | 3.55 | 3.47 | - | 13 |
| 7 | *PF3D7_0720400* |  | 3.53 | 3.79 | - | 4 |
| 7 | *PF3D7_0707200* |  | - | 3.58 | - | 3 |
| 7 | *PF3D7_0713500* |  | - | 3.87 | - | 4 |
| 7 | *PF3D7_0713600* |  | - | 3.54 | - | 6 |
| 7 | *PF3D7_0720700* |  | - | 3.45 | - | 2 |
| 7 | *PF3D7_0721000* |  | - | 3.79 | - | 4 |
| 7 | *PF3D7_0723800* |  | - | 3.82 | - | 2 |
| 8 | *PF3D7_0809200* | *pfa55-14* | 4.45 | 3.83 | 3.58 | 22 |
| 8 | *PF3D7_0809400* |  | 3.87 | 3.92 | 3.39 | 33 |
| 8 | *PF3D7_0809600* |  | 3.90 | 3.74 | 3.37 | 112 |
| 8 | *PF3D7_0830300* | *SIAP-2* | - | 4.05 | 3.56 | 4 |
| 8 | *PF3D7_0808100* |  | 3.74 | 3.57 | - | 3 |
| 8 | *PF3D7_0809700* | *RUVB1* | 3.95 | 3.51 | - | 22 |
| 8 | *PF3D7_0809800* |  | 3.58 | 3.60 | - | 13 |
| 8 | *PF3D7_0811700* |  | 3.48 | - | - | 2 |
| 8 | *PF3D7_0814500* |  | 4.06 | 3.58 | - | 7 |
| 8 | *PF3D7_0814600* |  | 3.56 | - | - | 5 |
| 8 | *PF3D7_0814700* |  | 3.80 | 4.11 | - | 12 |
| 8 | *PF3D7_0814900* | *FeSOD* | 3.67 | - | - | 2 |
| 8 | *PF3D7_0825700* |  | 3.76 | - | - | 4 |
| 8 | *PF3D7_0825800* |  | 3.60 | 3.68 | - | 5 |
| 8 | *PF3D7_0826000* |  | 3.79 | 3.68 | - | 30 |
| 8 | *PF3D7_0826100* |  | 3.76 | - | - | 7 |
| 8 | *PF3D7_0808200* |  | - | 3.77 | - | 6 |
| 8 | *PF3D7_0818600* |  | - | 3.58 | - | 3 |
| 8 | *PF3D7_0825500* |  | - | 3.40 | - | 2 |
| 9 | *PF3D7_0914000* |  | - | 3.87 | - | 2 |
| 10 | *PF3D7_1019600* |  | - | - | 3.21 | 5 |
| 11 | *PF3D7_1110200* | *PRPF6* | 3.95 | 4.44 | - | 6 |
| 11 | *PF3D7_1110300* |  | 3.68 | 3.49 | - | 2 |
| 11 | *PF3D7_1133400* | *AMA1* | 3.69 | 5.06 | - | 13 |
| 11 | *PF3D7_1126100* | *ATG7* | - | 3.95 | - | 4 |
| 13 | *PF3D7_1301800* | *SURFIN13.1* | - | 4.11 | 3.22 | 10 |
| 13 | *PF3D7_1318300* |  | 3.63 | - | - | 5 |
| 13 | *PF3D7_1335900* | *TRAP* | 3.53 | 5.13 | - | 26 |
| 13 | *PF3D7_1349100* |  | - | 3.88 | - | 2 |
| 13 | *PF3D7_1349200* |  | - | 3.48 | - | 2 |
| 13 | *PF3D7_1349500* |  | - | 3.86 | - | 4 |
| 13 | *PF3D7_1352400* |  | - | 3.82 | - | 2 |
| 13 | *PF3D7_1352600* |  | - | 3.40 | - | 2 |
| 13 | *PF3D7_1352700* |  | - | 3.58 | - | 4 |
| 13 | *PF3D7_1352900* |  | - | 4.34 | - | 32 |
| 14 | *PF3D7_1475900* |  | 3.69 | 3.87 | 3.13 | 16 |
| 14 | *PF3D7_1476600* |  | - | - | 3.20 | 2 |
| 14 | *PF3D7_1448500* |  | 3.73 | - | - | 7 |
| 14 | *PF3D7_1469600* | *ACC1* | 3.73 | - | - | 17 |
| 14 | *PF3D7_1475800* |  | 3.78 | 4.19 | - | 15 |
| 14 | *PF3D7_1462300* |  | - | 3.43 | - | 4 |
