# Supplementary material for: Imputation-Based Population Genetics Analysis of Plasmodium falciparum Malaria Parasites
Source: PLoS Genet. 2015 Apr 30;11(4):e1005131. doi: 10.1371/journal.pgen.1005131 (PMC4415759; doi:10.1371/journal.pgen.1005131)
Supplement: S6 Table — Median Rsb values per gene are shown. Only the 75 genes with 2 or more SNP hits across or within populations are shown. (DOCX) [file pgen.1005131.s017.docx]

**S6 Table.** Genes with SNPs in the top 1% of *Rsb* values in each population using complete-case haplotypes, with Malawi as the reference population. Median *Rsb* values per gene are shown. Only the 75 genes with 2 or more SNP hits across or within populations are shown.

| **Chr** | **Gene ID** | **Gene name** | **Thailand (Rsb)** | **Cambodia**  **(Rsb)** | **Gambia (Rsb)** | **Total SNPs** |
| --- | --- | --- | --- | --- | --- | --- |
| 2 | *PF3D7_0207400* | *SERA7* | - | 2.69 | 3.05 | 2 |
| 3 | *PF3D7_0304600* | *CSP* | 2.57 | 3.09 | 3.23 | 8 |
| 4 | *PF3D7_0405300* | *LISP2* | 2.54 | - | - | 6 |
| 5 | *PF3D7_0508900* |  | 2.75 | - | - | 5 |
| 5 | *PF3D7_0509400* | *RNAPI* | 2.74 | 2.54 | - | 4 |
| 7 | *PF3D7_0703900* |  | 2.81 | 2.55 | 2.97 | 12 |
| 7 | *PF3D7_0708200* |  | 3.02 | 3.06 | 2.95 | 9 |
| 7 | *PF3D7_0704100* |  | 2.60 | - | - | 2 |
| 7 | *PF3D7_0704300* |  | 2.86 | - | - | 7 |
| 7 | *PF3D7_0704400* |  | 2.57 | - | - | 2 |
| 7 | *PF3D7_0708400* | *HSP90* | 2.68 | 2.96 | - | 4 |
| 7 | *PF3D7_0708000* |  | - | 2.47 | - | 2 |
| 7 | *PF3D7_0708500* | *HSP86* | - | 2.75 | - | 3 |
| 7 | *PF3D7_0721200* |  | - | 2.48 | - | 3 |
| 7 | *PF3D7_0721500* |  | - | 2.71 | - | 6 |
| 10 | *PF3D7_1016500* |  | 2.70 | 3.38 | 2.87 | 9 |
| 10 | *PF3D7_1016700* |  | - | 2.70 | 2.97 | 8 |
| 10 | *PF3D7_1019700* |  | - | - | 3.41 | 2 |
| 10 | *PF3D7_1019600* |  | 2.64 | 2.79 | - | 8 |
| 10 | *PF3D7_1016300* | *GBP* | - | 2.69 | - | 7 |
| 12 | *PF3D7_1217200* |  | 3.07 | - | - | 2 |
| 12 | *PF3D7_1217300* |  | 2.82 | - | - | 3 |
| 12 | *PF3D7_1217400* |  | 2.82 | - | - | 2 |
| 12 | *PF3D7_1218200* |  | 2.72 | - | - | 4 |
| 12 | *PF3D7_1249800* |  | - | 2.45 | - | 5 |
| 13 | *PF3D7_1346400* |  | - | 2.80 | - | 2 |
| 13 | *PF3D7_1346500* |  | - | 2.81 | - | 2 |
| 13 | *PF3D7_1346700* | *P48/45* | - | 2.58 | - | 4 |
| 13 | *PF3D7_1346800* | *P47* | - | 2.73 | - | 6 |
| 13 | *PF3D7_1347900* |  | - | 2.56 | - | 2 |
| 13 | *PF3D7_1352900* |  | - | 2.65 | - | 7 |
| 13 | *PF3D7_1356400* |  | - | 2.49 | - | 2 |
| 13 | *PF3D7_1358200* |  | - | 2.52 | - | 3 |
| 14 | *PF3D7_1410300* |  | - | 2.53 | 3.41 | 11 |
| 14 | *PF3D7_1418100* |  | - | - | 3.10 | 3 |
| 14 | *PF3D7_1429400* |  | - | - | 3.42 | 2 |
| 14 | *PF3D7_1434500* |  | - | - | 2.99 | 11 |
| 14 | *PF3D7_1438400* | *MCA2* | - | - | 3.13 | 2 |
| 14 | *PF3D7_1442700* |  | - | - | 3.45 | 4 |
| 14 | *PF3D7_1445100* |  | - | - | 3.39 | 2 |
| 14 | *PF3D7_1446500* |  | - | - | 3.02 | 2 |
| 14 | *PF3D7_1446700* |  | - | - | 3.13 | 2 |
| 14 | *PF3D7_1447800* |  | - | - | 3.83 | 3 |
| 14 | *PF3D7_1447900* | *MDR2* | - | - | 4.07 | 4 |
| 14 | *PF3D7_1448000* |  | 2.62 | 2.46 | 4.09 | 3 |
| 14 | *PF3D7_1448500* |  | 2.67 | 2.57 | 3.65 | 25 |
| 14 | *PF3D7_1450800* |  | - | 2.41 | 3.93 | 2 |
| 14 | *PF3D7_1455600* |  | - | - | 3.06 | 2 |
| 14 | *PF3D7_1461800* |  | - | 2.61 | 4.04 | 6 |
| 14 | *PF3D7_1466200* |  | - | - | 2.97 | 2 |
| 14 | *PF3D7_1468900* |  | 2.62 | - | 3.27 | 5 |
| 14 | *PF3D7_1469600* | *ACC1* | 4.14 | 2.88 | 3.59 | 70 |
| 14 | *PF3D7_1469900* |  | 3.14 | - | 3.77 | 5 |
| 14 | *PF3D7_1470500* | *PDEdelta* | 2.73 | - | 3.35 | 6 |
| 14 | *PF3D7_1471200* | *SulP* | 2.73 | - | 3.53 | 4 |
| 14 | *PF3D7_1471400* |  | 2.70 | - | 4.05 | 5 |
| 14 | *PF3D7_1471600* |  | 3.02 | - | 3.29 | 5 |
| 14 | *PF3D7_1471800* |  | 3.02 | - | 2.93 | 7 |
| 14 | *PF3D7_1472200* |  | 2.84 | - | 3.39 | 3 |
| 14 | *PF3D7_1475400* | *CRMP4* | - | - | 3.18 | 2 |
| 14 | *PF3D7_1475800* |  | 2.68 | 2.47 | 3.95 | 21 |
| 14 | *PF3D7_1475900* |  | 2.81 | 3.22 | 4.27 | 44 |
| 14 | *PF3D7_1476200* |  | - | - | 2.90 | 3 |
| 14 | *PF3D7_1476600* |  | - | - | 2.92 | 8 |
| 14 | *PF3D7_1478900* |  | - | - | 3.32 | 4 |
| 14 | *PF3D7_1479000* | *ACS1a* | - | - | 3.32 | 3 |
| 14 | *PF3D7_1479100* |  | - | - | 3.27 | 3 |
| 14 | *PF3D7_1469200* | *SHLP1* | 2.55 | - | - | 3 |
| 14 | *PF3D7_1469800* |  | 2.95 | - | - | 2 |
| 14 | *PF3D7_1470100* |  | 2.94 | - | - | 15 |
| 14 | *PF3D7_1470700* |  | 2.81 | - | - | 5 |
| 14 | *PF3D7_1470800* |  | 2.63 | - | - | 2 |
| 14 | *PF3D7_1471300* |  | 2.91 | - | - | 4 |
| 14 | *PF3D7_1471700* |  | 3.17 | - | - | 5 |
| 14 | *PF3D7_1471900* |  | 2.72 | - | - | 5 |
