# Supplementary material for: Imputation-Based Population Genetics Analysis of Plasmodium falciparum Malaria Parasites
Source: PLoS Genet. 2015 Apr 30;11(4):e1005131. doi: 10.1371/journal.pgen.1005131 (PMC4415759; doi:10.1371/journal.pgen.1005131)
Supplement: S7 Table — Median Rsb values per gene are shown. Only the 103 genes with 2 or more SNP hits are shown. (DOCX) [file pgen.1005131.s018.docx]

**S7 Table.** Genes captured by the top 1% *Rsb* metrics in Cambodia using Beagle-imputed and complete-case haplotypes, with Thailand as the reference population. Median *Rsb* values per gene are shown. Only the 103 genes with 2 or more SNP hits are shown.

| **Chr** | **Gene ID** | **Gene**  **name** | **Cambodia**  ***Beagle* (Rsb)** | **Cambodia *complete***  **(Rsb)** | **Total SNPs** |
| --- | --- | --- | --- | --- | --- |
| 1 | *PF3D7_0103600* |  | 3.48 | - | 3 |
| 1 | *PF3D7_0104100* |  | 4.13 | - | 4 |
| 1 | *PF3D7_0113600* | *SURFIN1.2* | 5.15 | - | 40 |
| 1 | *PF3D7_0113800* |  | 5.19 | - | 73 |
| 2 | *PF3D7_0209000* | *P230* | 3.82 | - | 4 |
| 4 | *PF3D7_0419900* |  | 3.33 | - | 4 |
| 5 | *PF3D7_0504700* |  | 3.28 | 2.84 | 4 |
| 5 | *PF3D7_0504800* |  | - | 3.24 | 21 |
| 5 | *PF3D7_0505000* |  | - | 2.93 | 14 |
| 5 | *PF3D7_0532800* |  | 3.67 | - | 10 |
| 6 | *PBANKA_060370* |  | 3.96 | 2.85 | 3 |
| 6 | *PBANKA_060490* |  | - | 2.70 | 2 |
| 6 | *PBANKA_060520* |  | 3.33 | 3.07 | 2 |
| 6 | *PBANKA_061630* |  | - | 2.77 | 2 |
| 6 | *PBANKA_061640* |  | - | 2.84 | 2 |
| 6 | *PBANKA_060270* | *NAPL* | 3.44 | - | 2 |
| 6 | *PBANKA_060380* | *SEPSECS* | 4.41 | - | 3 |
| 6 | *PBANKA_060410* |  | 3.85 | - | 3 |
| 6 | *PBANKA_061740* |  | 3.70 | - | 8 |
| 7 | *PF3D7_0721000* |  | 3.66 | 2.78 | 13 |
| 7 | *PF3D7_0721200* |  | 3.50 | 3.41 | 12 |
| 7 | *PF3D7_0710200* |  | 3.45 | - | 3 |
| 7 | *PF3D7_0721300* | *DEAD/DEAH* | 3.36 | - | 10 |
| 7 | *PF3D7_0723700* |  | 3.38 | - | 13 |
| 7 | *PF3D7_0723800* |  | 3.46 | - | 17 |
| 7 | *PF3D7_0725100* |  | 4.08 | - | 2 |
| 8 | *PF3D7_0804500* |  | - | 3.04 | 2 |
| 8 | *PF3D7_0819800* |  | 3.37 | - | 2 |
| 8 | *PF3D7_0828400* | *Oxa1* | 5.39 | - | 2 |
| 8 | *PF3D7_0828500* |  | 4.05 | - | 2 |
| 8 | *PF3D7_0829900* |  | 3.54 | - | 8 |
| 8 | *PF3D7_0830100* |  | 4.00 | - | 3 |
| 8 | *PF3D7_0830300* | *SIAP-2* | 4.73 | - | 3 |
| 9 | *PF3D7_0926600* |  | - | 2.97 | 2 |
| 9 | *PF3D7_0913700* |  | 3.52 | - | 2 |
| 9 | *PF3D7_0913900* |  | 4.50 | - | 2 |
| 9 | *PF3D7_0914000* |  | 4.88 | - | 3 |
| 9 | *PF3D7_0916400* |  | 3.28 | - | 2 |
| 10 | *PF3D7_1019000* |  | 3.35 | - | 4 |
| 10 | *PF3D7_1035000* |  | 3.70 | - | 13 |
| 10 | *PF3D7_1035100* |  | 3.97 | - | 4 |
| 10 | *PF3D7_1035300* | *GLURP* | 3.59 | - | 3 |
| 11 | *PF3D7_1149000* | *Pf332* | - | 2.86 | 3 |
| 11 | *PF3D7_1149200* | *RESA* | 3.99 | 3.14 | 9 |
| 11 | *PF3D7_1149500* | *RESA2* | 3.55 | 2.90 | 10 |
| 11 | *PF3D7_1115300* | *FP2B* | 4.74 | - | 2 |
| 11 | *PF3D7_1126100* | *ATG7* | 3.50 | - | 4 |
| 11 | *PF3D7_1133400* | *AMA1* | 4.90 | - | 11 |
| 13 | *PF3D7_1342900* | *ApiAP2* | - | 3.42 | 13 |
| 13 | *PF3D7_1343600* |  | 3.42 | 2.80 | 2 |
| 13 | *PF3D7_1343700* | *PfK13* | 3.61 | 3.45 | 4 |
| 13 | *PF3D7_1343800* |  | 3.71 | 2.92 | 28 |
| 13 | *PF3D7_1344000* |  | 3.69 | 2.90 | 6 |
| 13 | *PF3D7_1347900* |  | 3.87 | 2.92 | 10 |
| 13 | *PF3D7_1348800* |  | 4.45 | 2.96 | 3 |
| 13 | *PF3D7_1349500* |  | 4.00 | 2.89 | 12 |
| 13 | *PF3D7_1350000* |  | 3.98 | 2.79 | 2 |
| 13 | *PF3D7_1350500* |  | 3.62 | 2.68 | 4 |
| 13 | *PF3D7_1350700* |  | 3.92 | 2.69 | 3 |
| 13 | *PF3D7_1350900* | *ApiAP2* | 4.20 | 4.26 | 2 |
| 13 | *PF3D7_1351000* |  | 4.39 | 2.82 | 6 |
| 13 | *PF3D7_1351200* |  | 3.84 | 2.79 | 4 |
| 13 | *PF3D7_1351300* |  | 3.29 | 2.90 | 2 |
| 13 | *PF3D7_1351700* | *ALV6* | 3.38 | 2.75 | 4 |
| 13 | *PF3D7_1351900* |  | 3.48 | 2.99 | 7 |
| 13 | *PF3D7_1352100* |  | 3.60 | 3.04 | 10 |
| 13 | *PF3D7_1352600* |  | 4.16 | 3.13 | 3 |
| 13 | *PF3D7_1356000* |  | 3.82 | 3.06 | 2 |
| 13 | *PF3D7_1356100* |  | 3.94 | 2.88 | 4 |
| 13 | *PF3D7_1356200* | *TIM23* | 4.30 | 2.88 | 2 |
| 13 | *PF3D7_1356400* |  | 5.08 | 2.74 | 5 |
| 13 | *PF3D7_1357700* |  | - | 2.71 | 3 |
| 13 | *PF3D7_1357800* |  | 3.47 | 2.70 | 2 |
| 13 | *PF3D7_1301800* | *SURF13.1* | 3.63 | - | 2 |
| 13 | *PF3D7_1306500* |  | 3.64 | - | 4 |
| 13 | *PF3D7_1308400* |  | 3.48 | - | 4 |
| 13 | *PF3D7_1335900* | *TRAP* | 4.24 | - | 44 |
| 13 | *PF3D7_1339700* |  | 3.49 | - | 2 |
| 13 | *PF3D7_1343400* | *RAD5* | 4.31 | - | 2 |
| 13 | *PF3D7_1348400* |  | 3.63 | - | 2 |
| 13 | *PF3D7_1349100* |  | 4.31 | - | 2 |
| 13 | *PF3D7_1352000* |  | 3.37 | - | 32 |
| 13 | *PF3D7_1352300* |  | 3.24 | - | 3 |
| 13 | *PF3D7_1352400* |  | 4.38 | - | 2 |
| 13 | *PF3D7_1352700* |  | 3.59 | - | 6 |
| 13 | *PF3D7_1352900* |  | 4.58 | - | 35 |
| 13 | *PF3D7_1353000* |  | 3.89 | - | 19 |
| 13 | *PF3D7_1353100* |  | 4.23 | - | 2 |
| 13 | *PF3D7_1353300* |  | 3.93 | - | 3 |
| 13 | *PF3D7_1354200* |  | 3.51 | - | 2 |
| 13 | *PF3D7_1356800* |  | 3.74 | - | 5 |
| 13 | *PF3D7_1357000* |  | 4.44 | - | 4 |
| 13 | *PF3D7_1357100* |  | 3.81 | - | 11 |
| 13 | *PF3D7_1357400* |  | 3.62 | - | 2 |
| 13 | *PF3D7_1358000* |  | 3.64 | - | 7 |
| 13 | *PF3D7_1359000* |  | 3.82 | - | 7 |
| 13 | *PF3D7_1359600* |  | 4.39 | - | 47 |
| 13 | *PF3D7_1359700* |  | 3.84 | - | 37 |
| 13 | *PF3D7_1362400* | *Pcalp* | 3.39 | - | 3 |
| 13 | *PF3D7_1364200* |  | 3.57 | - | 2 |
| 14 | *PF3D7_1475200* |  | - | 2.79 | 2 |
| 14 | *PF3D7_1462300* |  | 3.46 | - | 4 |
| 14 | *PF3D7_1462400* |  | 3.51 | - | 25 |
| 14 | *PF3D7_1475900* |  | 3.75 | - | 3 |
