# Supplementary material for: Imputation-Based Population Genetics Analysis of Plasmodium falciparum Malaria Parasites
Source: PLoS Genet. 2015 Apr 30;11(4):e1005131. doi: 10.1371/journal.pgen.1005131 (PMC4415759; doi:10.1371/journal.pgen.1005131)
Supplement: S8 Table — The diagonal contains the number of SNPs that are polymorphic in complete-case and post-imputation datasets. (DOCX) [file pgen.1005131.s019.docx]

**S8 Table.** Mean pairwise population *Fst* and 95% bootstrap confidence intervals using unimputed (lower triangle) and Beagle-imputed (upper triangle) genotypes. The diagonal contains the number of SNPs that are polymorphic in complete-case and post-imputation datasets.

|  | **Thailand** | **Cambodia** | **Gambia** | **Malawi** |
| --- | --- | --- | --- | --- |
| **Thailand** | 15,717/35,880 | 0.058  (0.056,0.059) | 0.259  (0.254,0.264) | 0.263  (0.257,0.269) |
| **Cambodia** | 0.057  (0.055,0.058) | 20,296/51,918 | 0.271  (0.265,0.276) | 0.267  (0.262,0.272) |
| **Gambia** | 0.260  (0.255,0.265) | 0.273  (0.268,0.278) | 13,097/32,920 | 0.040  (0.037,0.042) |
| **Malawi** | 0.263  (0.257,0.268) | 0.269  (0.263,0.274) | 0.035  (0.033,0.038) | 12,012/36,153 |
