# Supplementary material for: Imputation-Based Population Genetics Analysis of Plasmodium falciparum Malaria Parasites
Source: PLoS Genet. 2015 Apr 30;11(4):e1005131. doi: 10.1371/journal.pgen.1005131 (PMC4415759; doi:10.1371/journal.pgen.1005131)
Supplement: S9 Table — (DOCX) [file pgen.1005131.s020.docx]

**S9 Table.** The *P. falciparum* subtelomeric regions

| Chr. | End of 3' subtelomere | Start of 5' subtelomere |
| --- | --- | --- |
| 1 | 91652 | 565426 |
| 2 | 67544 | 860465 |
| 3 | 65507 | 1015545 |
| 4 | 170187 | 1150295 |
| 5 | 47760 | 1326241 |
| 6 | 39780 | 1329499 |
| 7 | 132430 | 1441012 |
| 8 | 58826 | 1349129 |
| 9 | 78873 | 1474420 |
| 10 | 61365 | 1599900 |
| 11 | 77999 | 2006704 |
| 12 | 56804 | 2190468 |
| 13 | 66918 | 2826696 |
| 14 | 28428 | 3266072 |
