# Supplementary material for: Correction: Imputation-Based Population Genetics Analysis of Plasmodium falciparum Malaria Parasites
Source: PLoS Genet. 2016 Aug 31;12(8):e1006300. doi: 10.1371/journal.pgen.1006300 (PMC5006977; doi:10.1371/journal.pgen.1006300)
Supplement: S2 File — (PDF) [file pgen.1006300.s002.pdf]

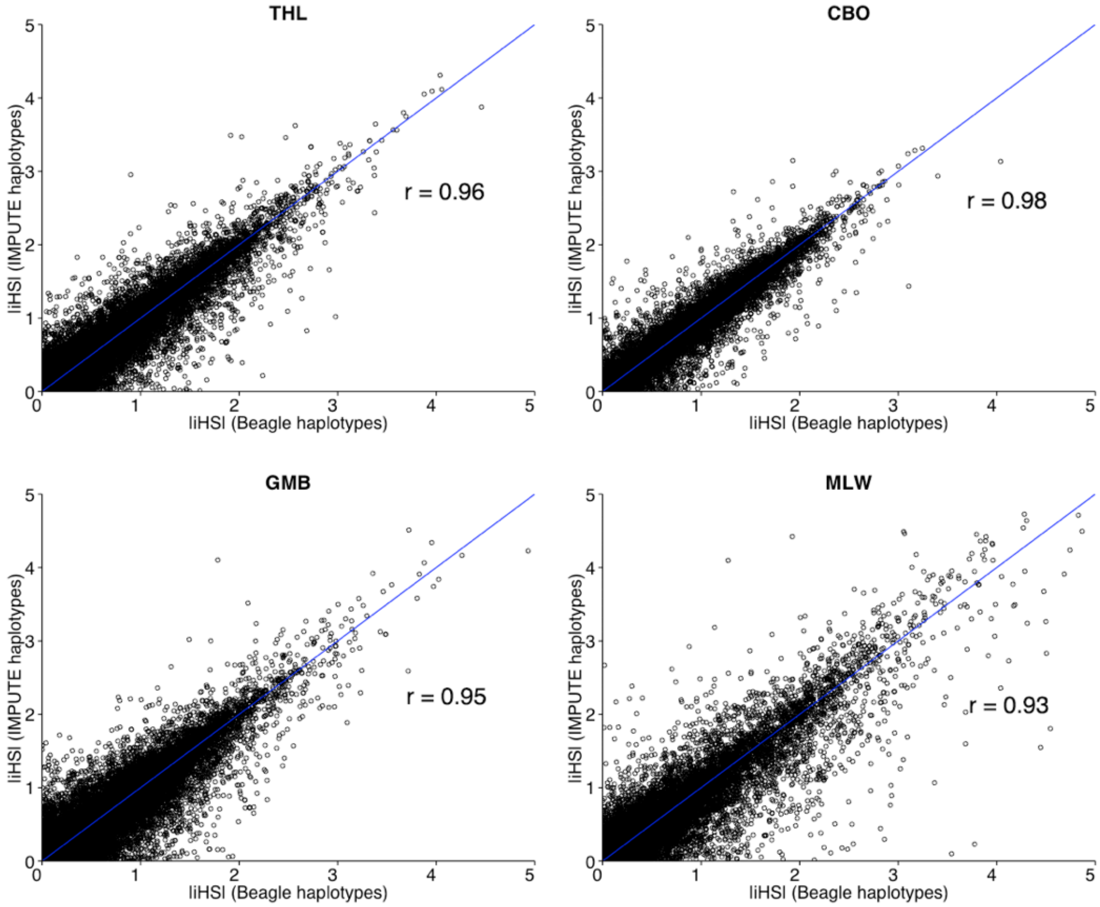

S. Figure 3. Pearson's correlation (*r)* between |*iHS*| metrics calculated from Beagle- or IMPUTE-imputed genotypes. Diagonal line indicates line of equality.
